# Recombinant *Limosilactobacillus* (*Lactobacillus*) delivering nanobodies against *Clostridium perfringens* NetB and alpha toxin confers potential protection from necrotic enteritis

**DOI:** 10.1002/mbo3.1270

**Published:** 2022-03-16

**Authors:** Dharanesh Gangaiah, Valerie Ryan, Daphne Van Hoesel, Shrinivasrao P. Mane, Enid T. Mckinley, Nallakannu Lakshmanan, Nandakumar D. Reddy, Edward Dolk, Arvind Kumar

**Affiliations:** ^1^ Division of Bacteriology and Microbiome Elanco Animal Health Greenfield Indiana USA; ^2^ Division of Nanobody Discovery and Development QVQ Holding BV Utrecht The Netherlands

**Keywords:** *Limosilactobacillus*, nanobodies, necrotic enteritis, poultry

## Abstract

Necrotic enteritis (NE), caused by *Clostridium perfringens*, is an intestinal disease with devastating economic losses to the poultry industry. NE is a complex disease and predisposing factors that compromise gut integrity are required to facilitate *C. perfringens* proliferation and toxin production. NE is also characterized by drastic shifts in gut microbiota; *C. perfringens* is negatively correlated with Lactobacilli. Vaccines are only partially effective against NE and antibiotics suffer from the concern of resistance development. These strategies address only some aspects of NE pathogenesis. Thus, there is an urgent need for alternative strategies that address multiple aspects of NE biology. Here, we developed *Limosilactobacillus* (*Lactobacillus*) *reuteri* vectors for in situ delivery of nanobodies against NetB and α toxin, two key toxins associated with NE pathophysiology. We generated nanobodies and showed that these nanobodies neutralize NetB and α toxin. We selected *L. reuteri* vector strains with intrinsic benefits and demonstrated that these strains inhibit *C. perfringens* and secrete over 130 metabolites, some of which play a key role in maintaining gut health. Recombinant *L. reuteri* strains efficiently secreted nanobodies and these nanobodies neutralized NetB. The recombinant strains were genetically and phenotypically stable over 480 generations and showed persistent colonization in chickens. A two‐dose *in ovo* and drinking water administration of recombinant *L. reuteri* strains protected chickens from NE‐associated mortality. These results provide proof‐of‐concept data for using *L. reuteri* as a live vector for delivery of nanobodies with broad applicability to other targets and highlight the potential synergistic effects of vector strains and nanobodies for addressing complex diseases such as NE.

## Graphical abstract

Necrotic enteritis is one of the most common diseases of poultry with significant economic losses. Here, we show that *Limosilactobacillus reuteri*‐delivered single domain antibodies (nanobodies) protect chickens from this disease.

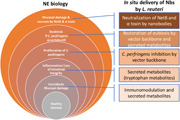

## 1 INTRODUCTION

Necrotic enteritis (NE) is a common intestinal disease that causes significant economic losses (~6 billion dollars annually) to the poultry industry worldwide (Cooper & Songer, 2009; Wade et al., 2015). NE generally manifests as clinical or subclinical forms. Clinical NE is acute and characterized by high mortality (30%–60%) and associated symptoms such as ruffled feathers, wet litter, diarrhea, and passage of undigested feed (Hofacre et al., 2018). In some cases, mortality is the only sign with no other premonitory symptoms. Subclinical NE is chronic and associated with damage to the intestinal mucosa, leading to reduced digestion and absorption of nutrients, decreased weight gain (3%–5%), and elevated feed conversion ratio (6–9 points) (Hofacre et al., 2018). Subclinical NE contributes to the majority of the economic losses associated with NE and is the most prevalent form of NE (Emami et al., 2021).

NE is caused primarily by *Clostridium perfringens* type A strains that infiltrate the mucosa of the small intestine and produce toxins such as NetB and α toxin (Al‐Sheikhly & Truscott, 1977a, 1977b; Emami & Dalloul, 2021; Emami et al., 2021; Hofacre et al., 2018; Justin et al., 2002; Keyburn et al., 2008; Kiu et al., 2019; Lee et al., 2012; Sheedy et al., 2004). *C. perfringens* is a spore‐forming, anaerobic, Gram‐positive commensal that is ubiquitously found in the gastrointestinal tract of animals and the environment. NE is a complex disease and several factors are known to influence the gut environment of the host and favor the growth of *C. perfringens* strains (Emami & Dalloul, 2021; Fernandes Da Costa et al., 2016). Mucosal damage caused by *Eimeria* species, nature of the feed, sudden diet change, high‐density bird housing conditions, and extreme environmental temperatures are among the key factors that predispose birds to NE (Fernandes Da Costa et al., 2016). The proliferation of *C. perfringens* leads to dramatic shifts in microbiota, with decreased abundance of beneficial species such as those belonging to *Lactobacillus* (Yang, Liu, Robinson, et al., 2021; Yang, Liu, Wang, et al., 2021).

The *C. perfringens* NetB is a pore‐forming toxin and it plays a key role in NE (Keyburn et al., 2008). A nontoxic variant of NetB called W262A is commonly used for immunization and has been shown to partially protect birds from NE (Fernandes Da Costa et al., 2016; Hunter et al., 2019; Jiang et al., 2015; Keyburn et al., 2008; S. Wang et al., 2021; Wilde et al., 2019). The role of α toxin in NE is not clear; however, immunization with α toxin antigen partially protects birds from NE (Abildgaard et al., 2009; Al‐Sheikhly & Truscott, 1977a, 1977b; Cooper et al., 2009; Fernandes Da Costa et al., 2016; Hunter et al., 2019; Jiang et al., 2015; Keyburn et al., 2006; Kulkarni et al., 2007, 2010; S. Wang et al., 2021; Wilde et al., 2019; Zekarias et al., 2008). A combination of NetB and α toxin provides improved protection against NE (Fernandes Da Costa et al., 2016). Nevertheless, vaccine approaches are only partially effective in reducing NE and the efficacy of vaccines depends on several factors such as the host genetics, immune system, and nutrition. To date, the administration of antibiotics has been the only effective treatment for NE; however, there is an increasing demand to reduce the use of antibiotics due to concerns around antimicrobial resistance. In addition, vaccines and antibiotics address only some aspects of NE (*C. perfringens* and their toxins), necessitating the need for developing safe and effective alternatives that address multiple aspects of NE biology.

Since their discovery in the 1990s, nanobodies (Nbs) have emerged as a promising alternative for disease prevention and treatment in both animal and human health (Amcheslavsky et al., 2021; Del Rio et al., 2019; Dulal et al., 2021; Hussack et al., 2014, Jovcevska & Muyldermans, 2020; Riazi et al., 2013; Steidler et al., 2000; Tremblay et al., 2013; Unger et al., 2015; Vanmarsenille et al., 2017; Zhang et al., 2021). Nbs are single‐domain antibodies, derived from heavy chain only (lack light chains and the first constant C_H_1 domain) antibodies that naturally occur in the serum of camelids (dromedaries, camels, llamas, alpacas, guanacos, vicunas) (Jovcevska & Muyldermans, 2020). The variable domain of these heavy‐chain only antibodies is the only domain involved in binding of this special class of antibodies (therefore called VHH, Variable domain of the Heavy chain of Heavy chain only antibodies) and are often referred to as “nanobodies,” which is a registered trademark of Ablynx. Compared to traditional antibodies, Nbs possess several unique and favorable properties such as small size, high stability, strong antigen‐binding affinity, water solubility, and ease of production in bacteria (Dumoulin et al., 2002; Goldman et al., 2017; Jovcevska & Muyldermans, 2020; Muyldermans et al., 2001, 2009; Van Der Linden et al., 1999; Van Der Vaart et al., 2006). Despite all these advantages, like any other protein and peptide biotherapeutics or preventatives, oral delivery of Nbs remains unsuccessful due to their degradation in the acidic and enzyme‐rich environment of the stomach (Dhalla et al., 2021; Gleeson et al., 2021). Desired efficacy also demands frequent administration at higher concentrations, which is not economically feasible.

Microbial vectors (MVs) offer an excellent opportunity for oral delivery of bio‐therapeutics and preventatives. MVs include bacteria such as *Limosilactobacillus* (*Lactobacillus*), *Lactococcus*, *Salmonella*, *Bacillus*, *Listeria*, and *Escherichia coli*, which are engineered to deliver target molecules directly to the site of action. Delivery using MVs not only protects the target molecules from the harsh gastrointestinal (GI) environment but also maximizes effectiveness and minimizes off‐target effects (Del Rio et al., 2019). MVs also have the advantage of easy and inexpensive manufacturing with flexible scalability and storage. Although several species of bacteria have been investigated as potential delivery vectors, species belonging to *Limosilactobacillus* and *Lactococcus* are among the most used genera for mucosal delivery of biotherapeutics and preventatives (Del Rio et al., 2019; Plavec & Berlec, 2019).

Lactobacilli are Gram‐positive bacteria that are mainly characterized by their ability to produce lactic acid from sugar and have been used to produce fermented products for decades. Many *Limosilactobacillus* species have “Qualified Presumption of Safety (QPS)” status from the European Food Safety Authority (EFSA) and “Generally Regarded As Safe (GRAS)” status from US Food and Drug Administration (FDA). Lactobacilli are key members of the endogenous microbiota of oral, GI, respiratory, and urogenital mucosa and play a key role in regulating local microbiota, restoring barrier function, preventing inflammation associated with GI diseases, improving growth performance, and protecting against infectious diseases (Chen et al., 2021; Han et al., 2021; Hu et al., 2021; Klaenhammer et al., 2002; Li et al., 2018; Manes‐Lazaro et al., 2017; Ouwehand et al., 2002; Siddique et al., 2021; Vineetha et al., 2017; Y. Wang et al., 2021; Wu et al., 2021). Of particular importance, Lactobacilli have been shown to possess strong antagonistic activity against *C. perfringens*, inhibit toxin production, reduce proinflammatory cytokines, improve intestinal integrity and immune response, correct microbial dysbiosis, restore performance deficiencies associated with subclinical NE, and protect chickens from clinical NE (Dec et al., 2016; Gong et al., 2020; Guo et al., 2017, 2021; Khalique et al., 2019; Kizerwetter‐Swida & Binek, 2005, 2009, 2016; La Cao et al.,  2012; La Ragione et al., 2004; Li et al., 2017; Shojadoost et al., 2022; Xu et al., 2020). Lactobacilli are also well known for surviving the harsh environment of the GI tract (Hai et al., 2021; Mandal et al., 2021; Noohi et al., 2021; Vesa et al., 2000). Furthermore, Lactobacilli are generally associated with mucosa and thus ensure delivery of target molecules directly to the mucosa. The availability of genetic tools for engineering *Limosilactobacillus* further makes them attractive candidates for in situ delivery of biomolecules.

Recombinant *Lactobacillus* have been widely used as live vectors to deliver therapeutic (cytokines, anti‐inflammatory, immunomodulatory and immunosuppressive molecules, growth factors, protease inhibitors) and prophylactic molecules (antigens) to treat and prevent various GI diseases, respectively (Alimolaei et al., 2017; Allain et al., 2016; Cano‐Garrido et al., 2015; Del Rio et al., 2019; Grangette et al., 2002; Ho et al., 2005; LeBlanc et al., 2013; Maassen et al., 1999; Mota et al., 2006; Steidler et al., 2003; M. Wang et al., 2016; Wu & Chung, 2007; Wyszynska et al., 2015; Xue et al., 2021). More specifically, *Lactobacillus* have been used as live delivery systems for Nbs targeting different infectious diseases and these applications have been extensively reviewed in Del Rio et al. (Andersen et al., 2016; Del Rio et al., 2019; Gunaydin et al., 2014; Kalusche et al., 2020).

In previous studies, we showed that two novel *Limosilactobacillus reuteri* (*L. reuteri*) isolates, ATCC PTA‐126787 (*L. reuteri* 3630) and ATCC PTA‐126788 (*L. reuteri* 3632) possess favorable safety properties based on the results from *in silico*, in vitro and in vivo analyses in chickens and Sprague Dawley rats (Gangaiah, 2021). In the present study, we describe the development of *L. reuteri* 3630 and *L. reuteri* 3632 as live vectors for in situ delivery of llama derived Nbs against *C. perfringens* NetB and α toxin to prevent NE in poultry. This study describes the application of *L. reuteri* delivered Nbs as a broad strategy to address a complex disease like NE.

## 2 MATERIALS AND METHODS

### 2.1 Llama immunization

A nontoxic variant of NetB (NetB W262A) and the C‐terminal fragment of α toxin (CPA_245–267_) were used for llama immunization (Fernandes Da Costa et al., 2016). Two llamas (SNL133 & SNL134) were immunized with 100 µg each of NetB and α toxin in four to six injections on Day 0, Day 14, Day 28, Day 35, Day 57, and Day 71. Blood samples were collected on Day 0, Day 28, Day 43, and Day 78 for analysis of immune response. Large bleeds were performed on Day 43 and Day 78 for RNA isolation and complementary DNA (cDNA) library preparation.

### 2.2 Analyses of immune response

The immune response of the llamas was tested in an ELISA coated with α toxin and NetB with the sera of llamas from Day 0, Day 28, Day 43, and Day 78. A MaxiSorp plate was coated with 200 ng antigen per well overnight at 4°C. After three times washing with phosphate‐buffered saline (PBS) containing 0.05% Tween‐20, the plate was blocked with 4% milk powder in PBS (MPBS). Next, a serial dilution of the sera in 1% MPBS was added to the wells and incubated for 1 h. Unbound antibodies were removed during washing with PBS. Subsequently, bound antibodies were detected with an anti‐VHH K1216 antibody and rabbit anti‐antibody coupled to peroxidase (Thermo Fisher). The binding of the antibodies was quantified by the colorimetric reaction of *O*‐phenylenediamine (OPD) in the presence of H_2_O_2_ at 490 nm.

### 2.3 Library construction

Immunization and RNA preparation were performed by Eurogentec (Belgium). Before use, the obtained RNA was precipitated and 5 µl (~100 ng) of RNA was loaded onto gel to confirm the integrity of 28S and 18S rRNA. The remaining RNA was stored in 70% ethanol, containing 200 mM sodium acetate at −80°C. About 40 µg RNA was transcribed into cDNA using SuperScript III Reverse Transcriptase Kit (Invitrogen) using commercial random hexamer primers (Thermo Fisher). The cDNA was cleaned on Macherey‐Nagel NucleoSpin Gel and PCR Clean‐up kit (Macherey‐Nagel). Immunoglobulin H (both conventional and heavy chain) fragments were amplified using primers annealing at the leader sequence region and the CH2 region as described previously (Dolk et al., 2012; Pardon et al., 2014). Five microliters were loaded onto a 1% Tris‐Borate‐EDTA (TBE) agarose gel to confirm amplification. The rest of the samples were loaded onto a 1% Tris‐Acetate‐EDTA (TAE) agarose gel. The 700‐bp fragment was excised from the gel and purified. About 80 ng was used as a template for the nested PCR. The amplified fragment was cleaned on Macherey‐Nagel NucleoSpin Gel and PCR Clean‐up kit and eluted in 120 µl. The eluted DNA was digested first with *Sfi*I and next with *Bst*EII. Restriction digestion was confirmed by agarose gel electrophoresis using 1.5% TBE agarose gel. After the restriction digestion, the samples were loaded onto a 1.5% TAE agarose gel. The 400‐bp fragment was excised from the gel and purified on Macherey‐Nagel NucleoSpin Gel and PCR Clean‐up kit. The 400‐bp fragments were ligated into the phagemid pUR8100 vector (QVQ BV) and transformed into *E. coli* TG1 (Nectagen). The transformed *E. coli* TG1 were titrated using 10‐fold dilutions. Five microliters of the dilutions were spotted on Luria–Bertani (LB) agar plates supplemented with 100 µg/ml of ampicillin and 2% glucose.

The number of transformants was calculated from the spotted dilutions of the rescued *E. coli* TG1 culture. The titer of the library was calculated by counting colonies in the highest dilution and using the formula below: library size = (amount of colonies) × (dilution) × 8 (ml)/0.005 (ml; spotted volume). The transformants were stored in a 2xYT (Sigma‐Aldrich) medium supplemented with 20% glycerol, 2% glucose, and 100 µg/ml ampicillin at −80°C. The insert frequency was determined by picking 24 different clones from transformations from each library and performing a colony PCR. Bands of ~700 bp indicate a cloned VHH fragment. Bands of ~300 bp indicate an empty plasmid.

### 2.4 Phage production and selection

Phages were produced from the libraries as outlined below (Dolk et al., 2012; Parmley & Smith, 1988; Smith, 1985). *E. coli* TG1 containing libraries from SNL133 (Days 43 and 78) and SNL134 (Days 43 and 78) were diluted from the glycerol stock up to an OD_600_ of 0.05 in 2xYT medium containing 2% glucose and 100 µg/ml ampicillin and grown at 37°C for 2 h to reach an OD_600_ of ~0.5. Subsequently, about 7 ml of the cultures were infected with helper phage VCS M13 using an MOI (multiplicity of infection) of 100 for 30 min at 37°C. *E. coli* TG1 were spun down and resuspended into 50 ml fresh 2xYT medium supplemented with both ampicillin (100 µg/ml) and kanamycin (25 µg/ml) and grown overnight at 37°C with shaking. Produced phages were precipitated from the supernatant of the cultures using polyethylene glycol (PEG)‐NaCl precipitation. Titers of the produced phages were calculated by serial dilution of the phage sample and infection of *E. coli* TG1.

Twenty microliters of the precipitated phages (~10^11^ phages, which is >1000‐fold the diversity of the libraries) were applied to wells coated with α toxin and NetB. In short, for each library, 100 µl antigen was coated on the MaxiSorp plate overnight at two concentrations of 5 and 0.5 µg/ml. As a negative control, one well was incubated with PBS only. The next day, after removal of non‐bound antigen, the plate was washed three times with PBS and blocked with 4% MPBS. At the same time, freshly precipitated phages were pre‐blocked with 2% MPBS for 30 min. Preblocked phages were incubated on coated antigen for 2 h. Upon extensive washing with PBS‐Tween and PBS, bound phages were eluted with 0.1 M triethylamine solution and subsequently neutralized with 1 M Tris/HCl, pH 7.5. Eluted phages were serially diluted and then used to infect *E. coli* TG1 bacteria and spotting on LB agar plates supplemented with 2% glucose and 100 µg/ml ampicillin and incubated overnight at 37°C.

Glycerol stocks were prepared from all outputs rescued by infection of *E. coli* TG1 and stored at −80°C. Simultaneously, TG1 cultures infected with the output of the selection on 5 µg/ml α toxin or NetB (highest coating) were used for phage production of SNL‐133 and SNL134 sublibraries to perform the second round of selection. Overnight grown rescued outputs were diluted 100‐fold in 5 ml fresh 2xYT medium supplemented with 2% glucose and 100 µg/ml ampicillin and grown for 2 h until log phase. Subsequently, 1 µl of helper phage VCS M13 was added and incubated at 37°C for 30 min. Cultures were allowed to produce phages overnight at 37°C. Produced phages were precipitated from the supernatant of the cultures using PEG‐NaCl precipitation. One microliter of the precipitated phages was applied to wells coated with α toxin or NetB as indicated below. Antigens were coated on a MaxiSorp plate overnight at three concentrations (5, 0.5, and 0.05 µg/ml) and phages that bind specifically to α toxin or NetB were identified as described in the first round of selection.

### 2.5 Screening and sequence analysis of VHHs

After the second round of phage display selection, glycerol stocks were prepared from all outputs rescued by infection of *E. coli* TG1 and stored at −80°C in the same way as for the outputs obtained after the first round of phage display selection. Subsequently, all rescued outputs of the second round of selection on both α toxin and NetB were plated out to pick single colonies, which were grown in a 96‐well plate (master plate EAT‐1 for α toxin and master plate ENB‐1 for NetB). These master plates were used to produce periplasmic fractions containing monoclonal VHHs for screening of binders.

The master plates were cultivated at 37°C in 2xYT medium supplemented with 2% glucose and 100 µg/ml ampicillin and stored at −80°C after the addition of glycerol to a final concentration of 20%. For the production of periplasmic fractions, master plates EAT‐1 and ENB‐1 were duplicated into a deep well plate containing 1 ml 2xYT medium supplemented with 0.1% glucose and 100 µg/ml ampicillin and grown for 3 h at 37°C before adding 1 mM isopropyl β‐d‐1‐thiogalactopyranoside (IPTG) for induction of VHH expression. The VHH expression was conducted overnight at room temperature. Periplasmic fractions were prepared by collecting the bacteria by centrifugation and their resuspension into 120 µl PBS. After freezing, bacteria were thawed and centrifuged to separate the soluble periplasmic fraction containing the VHH from the cell debris (pellet). To test the binding specificity, monoclonal VHHs were tested using 25 µl of the periplasmic fractions exactly as described above.

Based on the ELISA results, clones EAT‐1A2, EAT‐1F2, EAT‐1A3, EAT‐1F3, EAT‐1G3, EAT‐1G4, EAT1A6, EAT‐1E6, EAT‐1D7, and EAT‐1C8 from master plate EAT‐1 and clones ENB‐1A4, ENB‐1F4, ENB‐1B8, ENB‐1E8, ENB‐1B9, ENB‐1F10, ENB‐1D11, ENB‐1C12, and ENB‐1F12 from master plate ENB‐1 were selected for sequence determination.

### 2.6 Cloning, production, purification, and analysis of selected VHHs

From all the clones that were sequenced, EAT‐1A2, EAT‐1F2, EAT‐1A3, EAT‐1F3, EAT‐1G4, EAT1D7, and EAT‐1C8 from master plate EAT‐1 and ENB‐1A4, ENB‐1F4, ENB‐1B8, ENB‐1B9, ENB‐1F19, and ENB‐1D11 from master plate ENB‐1 were subcloned into an expression vector. VHH genes were cut out with *Sfi*I and *Eco*91I from phagemid pUR8100 into pMEK222 (QVQ BV) with the same sites. pMEK222 adds a c‐myc (EQKLISEEDL) and His‐tag (HHHHHH) at the C‐terminus of the VHH. The VHHs were produced as described below. Precultures were prepared by growing the bacteria containing the plasmids containing the selected VHH in 8 ml 2xYT medium supplemented with 2% glucose and 100 µg/ml ampicillin overnight at 37°C. The precultures were diluted into 800 ml fresh 2xYT that was pre‐warmed at 37°C and supplemented with 100 µg/ml ampicillin and 0.1% glucose. The bacteria were grown for 2 h at 37°C before induction of the VHH expression with 1 mM IPTG. The VHHs were expressed for 4 h at 37°C and bacteria were harvested by centrifugation. Bacterial pellets were resuspended into 30 ml PBS and frozen at −20°C.

Frozen bacteria were thawed at room temperature and centrifugated to separate cell debris and soluble fraction, which contains the VHH. VHH were purified from the soluble fraction using immobilized metal affinity chromatography resin charged with cobalt (TALON beads). Bound VHHs were eluted with 150 mM imidazole and dialyzed against PBS. The protein concentration was measured using absorption at 280 nm and corrected according to the molar extinction coefficient and the molecular weight of different VHHs. About 1 µg of the purified VHH was loaded onto sodium dodecyl sulfate‐polyacrylamide gel electrophoresis (SDS‐PAGE). The binding of purified VHH was analyzed by ELISA using immobilized α toxin or NetB as described above.

### 2.7 Neutralization of α toxin and NetB activity by selected nbs

#### 2.7.1 Neutralization of α toxin activity

The inhibitory capacity of the VHH antibodies directed toward α toxin was determined by measuring the α toxin lecithinase activity. Briefly, fresh egg yolk was centrifuged (10,000*g* for 20 min at 4°C) and diluted at 1:10 in PBS. The ability of the VHHs to neutralize the α toxin activity was assessed by preincubating a twofold dilution series of the VHHs with a constant amount of α toxin (either 5 µg/ml recombinant α toxin [C‐terminal fragment of α toxin used for llama immunization] or 3.33 × 10^−4^ U/µl α toxin from Sigma, P7633) for 30 min at 37°C before the addition of 10% egg yolk emulsion. As a control, serum from calves immunized with the recombinant α toxin (as used for llama immunization) was used, starting from a ¼ dilution. After incubation at 37°C for 1 h, the absorbance at 650 nm was determined. α Toxin activity was indicated by the development of turbidity which increases absorbance.

Neutralization of the α toxin hemolytic activity by the VHH antibodies directed toward α toxin was determined by measuring its effect on sheep erythrocytes. Similar to the inhibition of the α toxin lecithinase activity, the ability to neutralize the hemolytic activity was assessed by preincubating a twofold dilution series of the VHH antibodies with a constant amount of α toxin (6.25 × 10^−5^ U/µl α toxin from Sigma, P7633) for 30 min at 37°C before the addition of 1% sheep erythrocytes. No recombinant α toxin was used in this test because the recombinant toxin is not hemolytic. As a control, serum from calves immunized with the recombinant α toxin (as used for llama immunization) was used, starting from a ¼ dilution. After incubation at 37°C for 1 h, the plates were centrifuged to pellet intact red blood cells. The supernatants were transferred to a new 96‐well plate and the absorbance at 570 nm was determined. α Toxin activity was indicated by an increase in absorbance due to the release of hemoglobin from the erythrocytes.

#### 2.7.2 Neutralization of NetB activity

Neutralization of NetB hemolytic activity by VHH antibodies directed toward NetB was determined by measuring its effect on chicken erythrocytes. Similar to inhibition of α toxin activity, the ability to neutralize the NetB hemolytic activity was assessed by preincubating a twofold dilution series of the VHH antibodies with a constant amount of NetB toxin (20 µg recombinant NetB in a total volume of 2 µl) for 30 min at 37°C before the addition of 1% chicken erythrocytes. The nontoxic NetB variant W262A was included as well and showed no hemolysis. To understand the relative efficacy of Nb candidates compared to polyclonal antisera, serum from rabbits immunized with the recombinant NetB (wild type NetB, not the same as used for llama immunization) was used, starting from a ¼ dilution. After incubation at 37°C for 1 h, the plates were centrifuged to pellet intact red blood cells. The supernatants were transferred to a new 96‐well plate and the absorbance at 570 nm was determined. NetB activity was indicated by an increase in absorbance due to the release of hemoglobin from the erythrocytes.

### 2.8 Homology modeling and bioinformatics analyses of Nb clones and their evaluation for affinity, production, and stability

Four lead clones were selected and subjected to further optimization to improve affinity, production, and stability. Three‐dimensional (3D) structures of the Nb clones were generated using homology modeling as described previously (Khodabakhsh et al., 2021; Moonens et al., 2014). Based on the homology models and bioinformatics analyses, recombinant proteins were produced for the mutant clones and evaluated for affinity, production, and stability (protease and temperature) (Table 1). Trypsin digestion was used to assess protease stability. More specifically, VHH wild type and mutant clones were incubated with immobilized trypsin for different time points, 0, 15, 30, 45, 60, 90, 120, and 180 min at 37°C and resolved on an SDS‐PAGE gel. For temperature stability, melting temperatures were determined using Thermofluor assays using Sypro Orange at an Nb concentration of 0.5 mg/ml. Fluorescence was detected every 30 s using a LightCycler 480 at a heating rate of 0.5°C/min.

**Table 1:** Affinity, protease susceptibility, thermostability, and production levels of parent and mutant Nb clones

Target	Mutant clone	Sequence^a^	Estimated affinity (nM)	Trypsin susceptibility	Thermo‐stability (°C)	Production level (mg/L)
NetB	ENB‐IA4	EVQLVESGGGLVQAGGSLRLSCAASGSIFSTNVMGWYRQAPGKQREFVAGITIGGTA**R**YPDSVKGRFTISRDNTQNTVYLQMNNLKPEDTAVYYCNAVLPSDQRRWSWGQGTQVTVSS	0.7	Slightly susceptible to trypsin	*T*_m_ = 71.5	1.7
	ENB‐IA4_R57H	EVQLVESGGGLVQAGGSLRLSCAASGSIFSTNVMGWYRQAPGKQREFVAGITIGGTA**H**YPDSVKGRFTISRDNTQNTVYLQMNNLKPEDTAVYYCNAVLPSDQRRWSWGQGTQVTVSS	500	Improved trypsin resistance; however, lost affinity	*T*_m_ = 77.0	Increased production (13.4)
	ENB‐ID11	EVQLVESGGGLVQTGGSLRLSCTASGTIDMTYGLIWYRQAPGKERELVASIRRDG**R**TNYADSVKGRFTISIDNAKNSIHLQMNSLKPDDTARYYCNSPYHALWGQGTQVTVSS	0.5	Resistant to trypsin	*T*_m_ = 72.5	1.1
	ENB‐ID11_R56H	EVQLVESGGGLVQTGGSLRLSCTASGTIDMTYGLIWYRQAPGKERELVASIRRDG**H**TNYADSVKGRFTISIDNAKNSIHLQMNSLKPDDTARYYCNSPYHALWGQGTQVTVSS	0.3	Trypsin resistance similar to WT; retained affinity	*T*_m_ = 72.5	Increased production (10.1)
α Toxin	EAT‐IF2	EVQLVESGGGLVQAGGSLRLSCAGSG**R**TGSLYSMGWFRQAPGKEREFVAAITWRPSSTYYADSVKGRFTISRDDAKNTVYLQMNSLKPEDTAVYFCAARPRGGLSPTPQAYDYWGQGTQVTVSS	0.5	Trypsin susceptible	NT	0.3
	EAT‐1F2_R27H	EVQLVESGGGLVQAGGSLRLSCAGSG**H**TGSLYSMGWFRQAPGKEREFVAAITWRPSSTYYADSVKGRFTISRDDAKNTVYLQMNSLKPEDTAVYFCAARPRGGLSPTPQAYDYWGQGTQVTVSS	0.7	Improved trypsin resistance: retained affinity	NT	8.8
	EAT‐1F2_T28P	EVQLVESGGGLVQAGGSLRLSCAGSGR**P**GSLYSMGWFRQAPGKEREFVAAITWRPSSTYYADSVKGRFTISRDDAKNTVYLQMNSLKPEDTAVYFCAARPRGGLSPTPQAYDYWGQGTQVTVSS	0.5	Improved trypsin resistance; retained affinity	NT	11.9
	EAT‐IG4	EVQLVESGGGLVQPGGSLRLSCAASGSIATINDMGWFRQAPGKQRD**W**VATIVSDGSTAYADSVKGRFTISRDNAKNTVYLQMNSLKPEDTAVYYCSARRH**Y**GQGTQVTVSS	0.4	Slightly susceptible to trypsin	*T*_m_ = 66.0	0.9
	EAT‐1G4_W47L	EVQLVESGGGLVQPGGSLRLSCAASGSIATINDMGWFRQAPGKQRD**L**VATIVSDGSTAYADSVKGRFTISRDNAKNTVYLQMNSLKPEDTAVYYCSARRHYGQGTQVTVSS	70	Lost affinity; hence, not tested	*T*_m_ = 61.0	1.7
	EAT‐1G4_Y103W	EVQLVESGGGLVQPGGSLRLSCAASGSIATINDMGWFRQAPGKQRDWVATIVSDGSTAYADSVKGRFTISRDNAKNTVYLQMNSLKPEDTAVYYCSARRH**W**GQGTQVTVSS	2	Slightly lost affinity; hence, not tested	*T*_m_ = 68.0	4.3

Abbreviations: NT, not tested; *T*_m_, melting temperature.

^a^ The mutated amino acids are shown in bold.

### 2.9 In silico modeling to identify Nb‐binding epitopes on NetB

Nb binding epitopes on NetB were predicted by molecular docking method using MOE (Molecular Operating Environment), which helps to visualize, characterize, or evaluate the interaction of proteins with other proteins or ligands (Khodabakhsh et al., 2021). 3D structures of the Nb clones were generated using homology modeling. As a template, the crystal structure of NbFedF9 (PDB code: 4W6Y, 1.57A) was used (Moonens et al., 2014). One hundred models were generated by Modeller software version 9.24. The best model was selected based on discrete optimized protein energy score and the quality of the model was assessed by phi and psi analysis. The published crystal structure of NetB was used for in silico modeling (Savva et al., 2013).

### 2.10 Bacterial strains and growth conditions

Bacterial strains used in the present study are listed in Table 2. *L. reuteri* strains were propagated on *Lactobacilli* de Man Rogosa Sharpe (MRS; BD Difco) medium anaerobically at 37–39°C. *L. reuteri* strains with truncated *pyrE* were grown on MRS medium supplemented with 200 µg/ml uracil. *E. coli* strains were grown on LB or Brain Heart Infusion (BHI) medium aerobically at 37–39°C with shaking at 200 rpm. Where necessary, the growth media were supplemented with chloramphenicol at a final concentration of 25 µg/ml for *E. coli* and 15 µg/ml for *L. reuteri*.

**Table 2:** Bacterial strains and plasmids used in this study

Bacterial strain	Description	Source
*E. coli *strains		
DH5α	*E. coli* strain used for general cloning purposes	Invitrogen
TG1	A derivative of *E. coli* JM101; *E. coli* strain used for phage display	Lucigen
Plasmids		
pCG2440	A suicide vector backbone containing pE194 origin of replication, chloramphenicol resistance marker, and p15A origin of replication	This study
pVR01	pCG2440 suicide integration vector containing EAT‐1F2_R27H	This study
pVR02	pCG2440 suicide integration vector containing EAT‐1G4 (anti‐α toxin Nb)	This study
pVR03	pCG2440 suicide integration vector containing ENB‐1D11_R56H (anti‐NetB Nb)	This study
pVR04	pCG2440 suicide integration vector used for *pyrE* correction of NE08	This study
pVR05	pCG2440 suicide integration vector used for *pyrE* correction of NE12	This study
*L. reuteri*strains		
*L. reuteri* 3630 WT	*L. reuteri* strain isolated from chicken cecum	Gangaiah et al. (2021)
*L. reuteri* 3632 WT	*L. reuteri* strain isolated from chicken cecum	Gangaiah et al. (2021)
NE01	*L. reuteri* strain 3630 with intact *pyrE* delivering EAT‐1G4 (Nb against α toxin)	This study
NE06	*L. reuteri* strain 3632 with intact *pyrE* delivering ENB‐1D11_R56H (Nb against NetB)	This study
NE08	*L. reuteri* strain 3630 with truncated *pyrE* delivering EAT‐1G4 (Nb against α toxin)	This study
NE12	*L. reuteri* strain 3632 with truncated *pyrE* delivering ENB‐1D11_R56H (Nb against NetB)	This study
*C. perfringens*strains		
CP4	*C. perfringens* challenge strain used for efficacy evaluation	Southern Poultry Research Group Inc.
CP1‐1	*C. perfringens* strain isolated from clinical NE and positive for NetB and α toxin	Elanco Animal Health Inc.
JP1011	*C. perfringens* strain isolated from clinical NE and positive for NetB and α toxin	Elanco Animal Health Inc.

### 2.11 Antimicrobial activity analysis

*L. reuteri* strains 3630 and 3632 were evaluated for their ability to inhibit *C. perfringens* using an agar overlay method. Briefly, *L. reuteri* strains were streaked in the center of MRS agar plates and incubated overnight under anaerobic conditions at 37°C. The next day, *C. perfringens* was grown in BYC broth (BHI broth, 37 g/L; yeast extract, 5 g/L; l‐cysteine hydrochloride, 0.5 g/L) overnight under anaerobic conditions at 37°C was pelleted by centrifugation at 2000*g* for 10 min, washed twice in sterile PBS, and resuspended to an OD_600_ of 0.5. The culture was then added at 5% inoculum into molten agar cooled to 45°C, mixed well by swirling, and layered onto overnight grown *L. reuteri* plates. The plates were then incubated at 37°C for 24 h and observed for a zone of clearance around the *L. reuteri* streaks.

### 2.12 Untargeted metabolomics analyses

*L. reuteri* strains 3630 and 3632 were grown in AOF‐MRS (animal origin free‐MRS) broth supplemented with 0.5% galactooligosaccharides under anaerobic conditions at 37°C for 14–16 h. The cultures were then pelleted by centrifugation at 12,200*g*. The cell pellets were washed three times with ice‐cold PBS and the culture supernatants were filter‐sterilized; both the cell pellets and cell‐free supernatants were shipped to Metabolon for global untargeted metabolomics. The samples were analyzed for untargeted metabolomics exactly as described previously (Susanti et al., 2021).

### 2.13 Global proteomics analyses

#### 2.13.1 Sample processing

*L. reuteri* 3632 was grown overnight in MRS broth under anaerobic conditions at 37°C. The cultures were then pelleted by centrifugation at 12,000 *g*. The cell pellets were washed three times with ice‐cold PBS and the culture supernatants were filter sterilized. Both the cell pellets and cell‐free supernatants were shipped to MS Bioworks for global proteomics analyses as described below. Briefly, control media was passed over a Corning SpinX 6 ml 5 kDa molecular weight cut‐off (MWCO) filter spin column and concentrated/buffer exchanged against water to approximately 125 µl. The protein concentration of the sample was determined by Qubit fluorometry (Invitrogen) which reported a value of 6.15 µg/µl. Twenty microliters were loaded onto a gel for assessment.

The bacterial supernatant was concentrated to 900 µl on a 6 ml Corning SpinX, 5 kDa MWCO filter followed by 500 µl Corning SpinX 5 kDa MWCO, and the concentration determined by Qubit fluorometry which reported a value of 2.5 µg/µl. In total, 312.5 µg of total protein was recovered. The bacterial cell pellet was lysed in modified RIPA buffer (2% SDS, 150 mM NaCl, 50 mM Tris HCl pH 8.0) with mechanical disruption using a NextAdvance BulletBlender and 1.0 mm silica beads (3 min, setting 8). The protein concentration was determined by Qubit fluorometry which reported a value of 2.5 µg/µl. In all, 822 µg of total protein was recovered.

Twenty micrograms of each sample were processed by SDS‐PAGE using a 4%–12% Bis‐Tris NuPAGE mini‐gel (Invitrogen) with the MOPS (3‐(*N*‐morpholino)propane sulfonic acid) buffer system. Each gel lane was excised longitudinally into 20 equally sized bands and processed by in‐gel digestion with trypsin (Promega) using a ProGest robot (Digilab) with the protocol outlined below. Briefly, the excised gel lane was washed with 25 mM ammonium bicarbonate followed by acetonitrile, reduced with 10 mM dithiothreitol at 60°C followed by alkylation with 50 mM iodoacetamide at room temperature, digested with trypsin at 37°C for 4 h and finally quenched with formic acid and analyzed directly without further processing.

#### 2.13.2 Mass spectrometry

Half of each gel digest was analyzed by nano liquid chromatography with tandem mass spectrometry (MS/MS) with a Waters NanoAcquity HPLC system interfaced to a ThermoFisher Q Exactive. Peptides were loaded on a trapping column and eluted over a 75 µm analytical column at 350 nl/min; both columns were packed with Luna C18 resin (Phenomenex). The mass spectrometer was operated in data‐dependent mode, with the Orbitrap operating at 70,000 FWHM and 17,500 FWHM for MS and MS/MS, respectively. The 15 most abundant ions were selected for MS/MS. A total of 10 h of instrument time was used per sample.

#### 2.13.3 Data processing

Data were searched using a local copy of Mascot with the following parameters: Enzyme: Trypsin/P; Database: LREU3632_09182017. fasta (concatenated forward and reverse plus common potential contaminants); Fixed modification: Carbamidomethyl (C); Variable modifications: Oxidation (M), Acetyl (N‐term), Pyro‐Glu (N‐term Q), Deamidation (N/Q); Mass values: Monoisotopic; Peptide Mass Tolerance: 10 ppm; Fragment Mass Tolerance: 0.02 Da; Max Missed Cleavages: 2. Mascot DAT files were parsed into Scaffold (Proteome Software) for validation, filtering, and to create a nonredundant list per sample. Data were filtered at 1% protein and peptide false discovery rate (FDR) and we required at least two unique peptides per protein.

### 2.14 Construction of *pyrE*‐based integration vectors

All the plasmids and primers used in this study are listed in Tables 2 and 3, respectively. The vector pCG2440 was used to generate the integration vector and contains pE194 origin of replication (Horinouchi & Weisblum, 1982), p15A origin of replication for replication in *E. coli*, and chloramphenicol resistance marker for selection in both *E. coli* and *L. reuteri*. EAT‐1G4 and ENB‐1D11_R56H sequences were codon‐optimized to *L. reuteri* and synthesized from ATUM. Promoters and secretion signals were selected based on the global proteomics data and evaluated for their ability to express and secrete Nbs, respectively. Based on this analysis, the native promoter, secretion signal, and terminator from *cwlS* were chosen for further engineering to deliver Nbs. The expression cassette containing the *cwlS* promoter, *cwlS* secretion signal, codon‐optimized EAT‐1F2_R27H, and *cwlS* terminator was synthesized as a single fragment from GenScript Inc. pCG2440 backbone, 310 bases of 5′ flanking region containing 112 bp bases of *pyrE* upstream region and 198 bases of *pyrE* 5′ coding region, the expression cassette containing EAT‐1F2_R27H and 1200 bases downstream of *pyrE* CDS were PCR amplified using P1, P2, P3, and P4 primer pairs, respectively (Table 3), and NEB 2X Phusion master mix following the manufacturer's instructions. The PCR products were treated with DpnI (Thermo Fisher Scientific) to digest the vector backbone following the manufacturer's instructions. The digested products were PCR purified using a Qiagen PCR Purification kit (Qiagen) and assembled to generate pVR01 using Gibson Assembly kit (NEB) following the manufacturer's instructions. The assembled product was transformed into chemically competent *E. coli* cells provided in the kit and plated on BHI agar containing 25 µg/ml of chloramphenicol to select for transformants. The transformants were confirmed for the presence of all the fragments by PCR using NEB Phusion 2X master mix (NEB) and plasmid DNA was isolated from select clones using Qiagen Midi Prep Plasmid Isolation kit (Qiagen) following the manufacturer's instructions. Similarly, pVR02 and pVR03 were generated by replacing the EAT‐1F2_R27H sequence in pVR01 with codon‐optimized EAT‐1G4 and ENB‐1D11_R56H sequences using P5‐P8 primer pairs and molecular biology procedures described above (Table 3).

**Table 3:** Primers used in the present study

Primer name	Sequence 5′–3′	Description
P1F	agtcgacttttttttcgg	Amplify pCG2440 backbone to generate pVR01
P1R	aaattgatctccttccatc	
P2F	agatggaaggagatcaatttgaatagatgggagcaatg	Amplify 5′ homologous region to generate pVR01
P2R	cttttaatttctcactaaaatgaagtttaatttg	
P3F	ttttagtgagaaattaaaaggctggattttttc	Amplify expression cassette to generate pVR01
P3R	taattaattacaatttgtctccctcataaaac	
P4F	gagacaaattgtaattaattatcaaaaaagcctaagattg	Amplify 3′ homologous region to generate pVR01
P4R	gcccgaaaaaaaagtcgactcaccaaacaccatttatg	
P5F	tacttccctggaaattactatg	Amplify pVR01 backbone to generate pVR02
P5R	tgtttcatttttatctttggttg	
P6F	ccaaagataaaaatgaaacagaagttcaacttgtagagtcc	Amplify EAT‐IG4 to generate pVR02
P6R	tagtaatttccagggaagtacgctgcaccatgatgatg	
P7F	tacttccctggaaattactatg	Amplify pVR01 backbone to generate pVR03
P7R	tgtttcatttttatctttggttg	
P8F	ccaaagataaaaatgaaacagaagtacaacttgttgaatcc	Amplify EAT‐IG4 to generate pVR03
P8R	tagtaatttccagggaagtaagctgcaccatgatgatg	
P9F	aaattaaaaggctggattttttc	Amplify pVR02 backbone to generate pVR04
P9R	ctcactaaaatgaagtttaatttg	
P10F	ttaaacttcattttagtgaggccgatgttattgccggaac	Amplify *pyrE* correction insert to generate pVR04
P10R	aaaatccagccttttaatttttatgatgcttgctctttactcc	
P11F	aaattaaaaggctggattttttc	Amplify pVR03 backbone to generate pVR05
P11R	ctcactaaaatgaagtttaatttg	
P12F	ttaaacttcattttagtgaggccgatgttattgccggaac	Amplify *pyrE* correction insert to generate pVR05
P12R	aaaatccagccttttaatttttatgatgcttgctctttactcc	

### 2.15 Transformation of integration plasmids into *L. reuteri*

*L. reuteri* strains were grown in 45 ml of MRS broth containing 1% glycine and 2% maltose (pH 6.5) for 12–14 h at 39°C in an anaerobic chamber. The cells were incubated on ice for 10 min, pelleted by centrifugation at 2000 *g* for 10 min, and washed twice with 30 ml of ice‐cold water. The washed cells were resuspended in 5 ml of ice‐cold 0.5 M EDTA solution and incubated on ice for 5 min. Following incubation, the cells were pelleted and washed twice with 30 ml of ice‐cold transformation buffer (0.33 M sucrose, 10% glycerol). Washed cells were resuspended in 300 µl of ice‐cold transformation buffer (0.33 M sucrose and 10% glycerol) and aliquoted into 50 µl aliquots in sterile 1.5 ml tubes. The integration plasmids were transformed into *L. reuteri*‐competent cells in a 2 mm electroporation cuvette (2.5 KV, 25 μF, 200 Ω, 2 mm). Immediately after electroporation, 900 µl of pre‐warmed MRS broth was added and incubated in an anaerobic chamber at 39°C for at least 4 h. Following incubation, the cells were plated on MRS (BD Difco) with 15 µg/ml of chloramphenicol and incubated for 2–5 days in an anaerobic chamber at 39°C. Transformants were selected, and streak purified three times before proceeding to the next steps.

### 2.16 Selection of single crossovers and their resolution into double crossovers

To utilize PyrE as a counterselection marker for selecting double crossover integrants, it is imperative that we select single crossover integrants at 3′ end with functional uracil pathway (Sakaguchi et al., 2013). Transformants with a functional PyrE will not grow in the presence of 5‐fluoroorotic acid (5‐FOA) (Sakaguchi et al., 2013). The streak purified colonies were plated on MRS containing 1 mg/ml of 5‐FOA. Three colonies that did not grow on AOF‐MRS containing 1 mg/ml of 5‐FOA were selected for further resolving into double crossovers. The selected colonies were subcultured three times in MRS media containing 200 µg/ml uracil and plated on AOF‐MRS containing 1 mg/ml of 5‐FOA and 200 µg/ml uracil and grown overnight in the anaerobic chamber at 39°C. The double crossover colonies were streak purified on AOF‐MRS containing 1 mg/ml of 5‐FOA and 200 µg/ml uracil three times, the colonies were PCR and sequence confirmed, and freezer stocks were prepared for the final clones. The final confirmed double crossovers with truncated *pyrE* delivering EAT‐1G4 (α toxin) and ENB‐1D11_R56H (NetB) Nbs were named NE08 and NE12, respectively.

### 2.17 Construction of *pyrE*‐based correction vectors and their integration into *L. reuteri* genome

In this step, the truncated *pyrE* was corrected to its wild‐type state using *pyrE* correction vectors. PyrE was reconstituted by PCR amplification and assembly of pVR02 backbone and 444 bp of the 3′ end of *pyrE* gene using primer pairs P9 and P10, respectively, generating pVR004, using the molecular biology procedures described above (Table 3). Similarly, pVR05 was generated by reconstituting *pyrE* using pVR03 as backbone and primer pairs P11–P12 (Table 3). Transformation of correction plasmids was performed exactly as described above except that the transformants were plated on MRS with 15 µg/ml of chloramphenicol and 200 µg/ml of uracil and the streak purified transformants were plated on AOF‐MRS containing 1 mg/ml of 5‐FOA and 200 µg/ml of uracil. Transformants with 3′ integration will grow well on MRS AOF‐MRS containing 1 mg/ml of 5‐FOA and 200 µg/ml of uracil due to the nonfunctional uracil pathway. The 3′ single crossover integrants were resolved into double crossovers as described below. The transformants were subcultured twice in MRS media containing 200 µg/ml uracil followed by a third culture in modified DMEM media with no uracil. After the third subculture, the cells were plated on AOF‐MRS with reduced uracil and incubated at 39°C for 24 h. The final double crossover colonies were streak purified three times and robustly growing colonies were PCR and sequence confirmed for the presence of intact *pyrE* and the expression cassette. The final confirmed double crossovers with intact *pyrE* delivering EAT‐1G4 (α toxin) and ENB‐1D11_R56H (NetB) Nbs were named NE01 and NE06, respectively.

### 2.18 Ammonium precipitation, SDS‐PAGE, and western blot analyses

NE01 and NE06 were grown in buffered AOF‐MRS media overnight at 39°C under anaerobic conditions. Following growth, the culture was centrifuged at 12,200 *g* for 10 min at 4°C and the supernatant was collected, filter sterilized using a 0.22 µm syringe filter, and pH adjusted to 7.0. Ammonium sulfate was added to the sample to achieve 70% saturation and incubated overnight at 4°C. The following day, the precipitated proteins were pelleted by centrifugation at 20,000 *g* for 30 min at 4°C. The pellet was resuspended to 5% of the original volume in 1 × DPBS and stored at −20°C until use. An aliquot of the precipitated sample was treated with sample buffer (LSD Sample Buffer; Novex) and separated on SDS‐PAGE (Bolt 4%–12% Bis–Tris Plus gel) and analyzed by western blot using MonoRab Rabbit Anti‐Camelid VHH Cocktail primary antibody (GenScript Inc.) and IRDye 800CW Goat anti‐Rabbit secondary antibody (LiCor).

### 2.19 Stability analyses of engineered *L. reuteri* strains

NE01 and NE06 were passaged for 30 passages as described below. A colony was streaked for single colony isolation on MRS agar plates supplemented with 200 µg/ml uracil where needed, the plates were incubated under anaerobic conditions overnight at 39°C, a single colony was picked, and further re‐streaked for single colony isolation. This process was repeated an additional 29 times. Passage 30 was scraped from the agar plates and suspended in 1 ml of AOF‐MRS media containing 20% glycerol and stored at −80°C. The final passage strains were confirmed for intactness of the expression cassette and flanking regions by Sanger sequencing and whole‐genome sequencing, and for secretion of Nbs by western blot analyses using procedures described above.

### 2.20 Neutralization of NetB activity by *L. reuteri* secreted Nbs

NE06 was grown overnight under anaerobic conditions at 37°C and Nbs were precipitated from overnight cultures by ammonium precipitation as described above. The ammonium precipitated proteins were further purified via IMAC using a 5 ml HisTrap Fast Flow Crude column (GE). The purified Nbs were tested for anti‐NetB activity using fresh chicken red blood cells as described above.

### 2.21 Colonization of engineered *L. reuteri* strains

*L. reuteri* candidates NE01 and NE06 along with their respective parent strains, *L. reuteri* 3630 and *L. reuteri* 3632, were evaluated for colonization in White Leghorn (Charles River Laboratories), specific pathogen‐free (SPF) chickens following in ovo administration. The strains were marked with rifampicin resistance using metabolic drift mutant (MDM) technology to selectively isolate these strains from the rest of the gut microbiota. To generate MDM clones, the parent strains were plated on different concentrations of rifampicin, and naturally occurring resistant mutants were selected and passaged for 160 generations to confirm the stability of resistance. The colonization study was conducted at Southern Poultry Research Group Inc. and the study was reviewed and approved by Elanco Animal Health Inc., Animal Care and Use Committee. Briefly, at 18 day of embryonation, 75 embryonated eggs were randomly assigned into five groups with 15 eggs in each group. Groups 1 and 2 were inoculated by amniotic route with 1 × 10^7^ colony‐forming units (CFUs)/embryo of NE01 and NE06, respectively. Groups 3 and 4 were inoculated by amniotic route with 1 × 10^7^ CFUs/embryo of *L. reuteri* 3630 and *L. reuteri* 3632, respectively. Group 5 served as a control and was inoculated with Marek's disease vaccine diluent. After hatching, on Day 3 and Day 7, cecal samples were collected, homogenized, serially diluted, and plated on MRS agar with 50 µg/ml of rifampicin. The plates were incubated under anaerobic conditions at 37°C for 24 h and CFUs were quantified.

### 2.22 Efficacy evaluation of NE01 and NE06 in an NE model

#### 2.22.1 Study design

The efficacy study was conducted at Southern Poultry Research Group Inc. and the study was reviewed and approved by Elanco Animal Health Inc., Animal Care and Use Committee. Cobb 700 broiler chicken eggs on the 18th day of embryonation were allocated to six groups with each containing at least 180 embryonated eggs. On the day of hatch, chicks from each group were divided into 18 replicates each containing 10 chicks (180 total). Chicks from each replicate were housed in the same cage and replicates of the same group were housed on the same rack. The birds were housed and cared for according to the Guide for the Care and Use of Agricultural Animals in Research and Teaching. The birds were provided with ad libitum feed and drinking water. The feed ration included a commercial‐type broiler diet formulated to meet or exceed requirements stipulated by the National Research Council (Council, 1994).

#### 2.22.2 Preparation of *L. reuteri* candidates and their administration

Seed cultures for fermentation were prepared by adding a loopful of freezer stock of NE01 and NE06 (not resistant to rifampicin) into a 250 ml vented flask with 50 ml of MRS medium and incubated overnight at 39°C under anaerobic conditions. The following day, the seed cultures were added to a fermenter with 5 L of MRS medium and grown for up to 8 h. The experimental product was prepared by adding an equal volume of bacterial culture and stabilizer, aliquoted and lyophilized. Viable cell counts (the numbers of CFUs) were determined by plating serial dilutions onto MRS agar plates. The vials containing the experimental products were tested for purity using a modified 9 CFR 113.64 protocol.

*L. reuteri* candidates were administered as a two‐dose vaccine scheme with the first dose being administered by in ovo to the 18th day of embryonation eggs or coarse spray to day‐old chicks and the second dose administered to 13‐day‐old chicks via drinking water. For administration at each timepoint, vials containing the experimental product were resuspended with the appropriate volume of nonchlorinated distilled water. Following resuspension, an aliquot was used for titration. Embryonated eggs in the challenge control group were injected with 0.1 ml of Marek's disease vaccine diluent. Embryonated eggs in the in ovo‐treated groups were injected with 0.1 ml of the appropriate dose of the NE01 and NE06 products. On the day of hatch, chicks designated for spray administration were kept in a chick basket and sprayed with the appropriate volume of resuspended *L. reuteri* using a hand‐held sprayer. Each chick received approximately 0.25 ml/chick of the NE01 and NE06 products. For drinking water administration, water was withheld for all chickens for approximately 2 h before administration of the experimental product. Following the withdrawal period, the resuspended experimental product was added to the waterer before chickens were allowed to drink. Immediately after water containing the *L. reuteri* was consumed, fresh drinking water, without *L. reuteri*, was added to the waterer for ad libitum consumption.

#### 2.22.3 Challenge with *Eimeria maxima* and *C. perfringens*

The NE model used in the present study includes oral administration of *E. maxima* followed by serial oral administration of *C. perfringens*. At Day 14 of age, the birds in Groups 2, 3, 4, and 5 were challenged with 25,000 sporulated oocysts of *E. maxima*/bird. The challenge inoculum was administered via oral gavage using a 10 ml syringe fitted with an 18‐Gauge gavage needle.

Fresh *C. perfringens* (strain CP4) challenge inoculum was prepared every day from a stock culture in fluid thioglycollate broth overnight at 35°C under anaerobic conditions. *C. perfringens* was administered by oral gavage at a target dose of approximately 1 × 10^8^ CFUs/ml (1 ml/chick) on 18, 19, and 20 days of age.

#### 2.22.4 Assessment of protection

Mortality associated with NE was used as the primary criteria for evaluating the efficacy of NE01 and NE06 candidates. Chickens that died postchallenge phase between 18 (after challenge with *C. perfringens*) and 28 days of age were necropsied, and the cause of death was listed as NE‐related or non‐NE‐related mortality.

### 2.23 Statistical analyses

The in vitro and colonization data presented in this study were analyzed using a mixed model analysis of variance with experimental days or chicks as a random effect followed by Tukey's test for pairwise multiple comparisons where applicable. A *p* value of 0.05 was considered statistically significant.

Statistical analyses of metabolomics data were performed in ArrayStudio on log‐transformed data. Raw intensity values were rescaled for each identified metabolite by dividing them by the median intensity across samples. For all analyses, missing values, if any, were imputed with the observed minimum for that compound. Secreted metabolites were identified by comparing the scaled and imputed intensities to the respective metabolites in media controls. A 1.5‐fold increase in scaled intensities over media was used to define metabolites secreted.

For efficacy study, data available from previous efficacy studies were used to perform power calculations to determine the number of chickens enrolled into each group. Statistical comparisons of the NE‐associated mortality were conducted via a two‐sided test at the α level set to 0.05. No multiplicity adjustment was applied. Statistical analysis was performed using SAS PROC FREQ and PROC MIXED with the ddfm=KR option. The analysis of NE‐induced mortality from Study Day 18 to Study Day 28 assumed that the cage effect is zero. For the comparison of Groups 1 versus 2, Fisher's exact test was used due to zero mortality incidence in Group 1. For other comparisons, the likelihood ratio *χ* ^2^ test was used. The mortality rate was reported for each treatment group. Preventative fractions were calculated by subtracting the mortality in Groups 3, 4, and 5 from Group 2, and expressing the difference in mortality as a proportion of the latter.

## 3 RESULTS

### 3.1 Llama immunization and selection of NetB‐ and α toxin‐specific nbs

**Figure 1:**
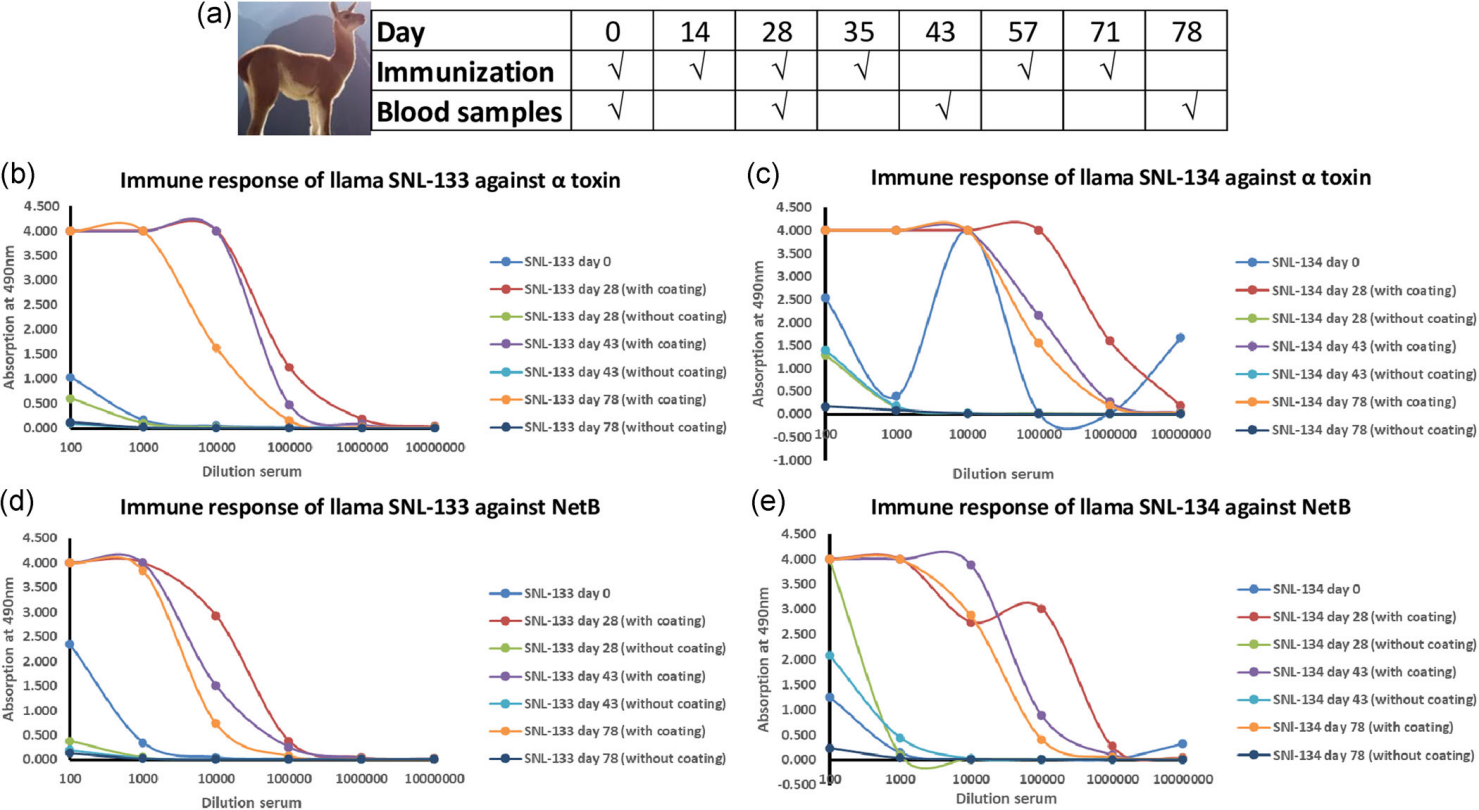
Llama immunization scheme (a) and immune response of SNL‐133 (b and d) and SNL‐134 (c and e) to directly coated α toxin (b and c) and NetB (d and e). Two llamas, SNL133 and SNL134, were immunized with NetB and α toxin toxoids on Day 0, Day 14, Day 28, Day 35, Day 57, and Day 71, and sera were collected at different time points and analyzed for an immune response using ELISA. Representative results are shown from three independent experiments

As shown in Figure 1a, two llamas (SNL133 and SNL134) were immunized with NetB and α toxin toxoids on Day 0, Day 14, Day 28, Day 35, Day 57, and Day 71. Immune response analysis showed that both llamas had a high immune response to both antigens (Figure 1b–e). None of the sera gave background on the noncoated wells. Similarly, sera of both llamas from Day 0 showed no binding, except for one dilution for SNL‐134 on α toxin (Figure 1b–e). For both llamas on both antigens, the sera of Day 28 seemed to give a higher response than the sera of both Day 43 and Day 78 (Figure 1b–e), suggesting that the titer of antibodies already reached a maximum at Day 28 of the immunization. Although the antibody population in the sera on Day 43 and Day 78 was not higher than Day 28, we reasoned that the antibodies present in the sera on Day 43 and Day 78 could be more maturated and hence beneficial in selecting Nbs specific to NetB and α toxin. Overall, a good immune response was observed against both toxoids in both llamas.

RNA was isolated from the major bleeds on Day 43 and Day 78 and cDNA libraries were prepared. As shown in Table B1, all four libraries (two for NetB on Days 43 and 78; two for α toxin on Day 43 and 78) were of good size with more than 10^7^ clones per library, which was sufficient for efficient panning selections. Analysis of insert frequency showed that the frequency was close to 100% for all four libraries.

A total of four phage libraries were constructed, and two rounds of panning, selection, and screening were performed. In the first round of panning, each library showed an output of eluted phages binding to each antigen. Each library showed almost no background of nonspecific binding phages on the noncoated wells. For α toxin, lower antigen concentration resulted in a lack of output of the binding phages. For NetB, a concentration dependence was observed in the outputs. For both antigens, the output eluted from the wells coated with 5 µg/ml of antigen looked promising. Similarly, in the second round of panning, all the libraries showed no nonspecific binding phages eluted from the noncoated wells at the same dilution used for antigen. All libraries showed a high output of binding phages eluted from the coated wells. All outputs showed a concentration‐dependent enrichment in the eluted phages between different concentrations of the wells coated with antigen. The high outputs from the second round of panning compared to the smaller outputs of the first round of panning suggest that the eluted binding phages bind specifically to the antigen.

**Figure A1:**
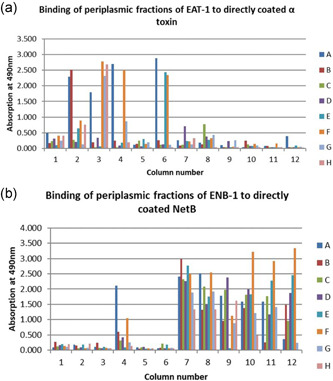
Binding of the periplasmic fractions of the master plate EAT‐1 to α toxin (a) and binding of the periplasmic fractions of the master plate ENB‐1 to NetB (b). The binding strength is indicated by absorbance at 490 nm

Following the second round of phage display selection, master plates of monoclonal VHH were picked, and periplasmic fractions containing monoclonal VHHs were extracted and screened for binding specificity to NetB and α toxin. As shown in Figure A1, there were several clones from master plate EAT‐1 that were bound to α toxin. Most of the binders were selected from library SNL‐133, Day 43. Similarly, there were several binding clones from master plate ENB‐1 that were bound to NetB (Figure A1). Most of these binding VHHs were derived from the selection with libraries SNL‐133, Day 78 and SNL‐134, Day 78.

**Figure A2:**
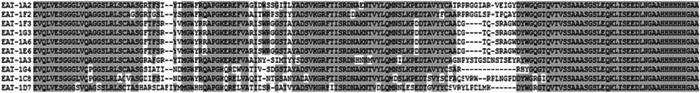
Alignment of the sequences of the VHHs picked from master plate EAT‐1. Conserved residues are highlighted

**Figure A3:**
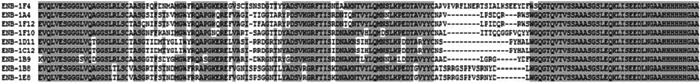
Alignment of the sequences of the VHHs picked from master plate ENB‐1. Conserved residues are highlighted

Based on the ELISA results, several clones were picked from master plates EAT‐1 and ENB‐1 for sequence identification. Figure A2 shows the sequence alignment of the clones that were picked from the selection outputs on α toxin. Two families are shown (KEREF and KQREL) within the sequences. There is a diversity of around seven different VHH sequences. Figure A3 shows the sequence alignment of the clones that were picked from the selection outputs on NetB. There is a diversity of around 6 different VHH sequences, which were derived from two families (KEREF and KQREL) within the sequences.

**Figure 2:**
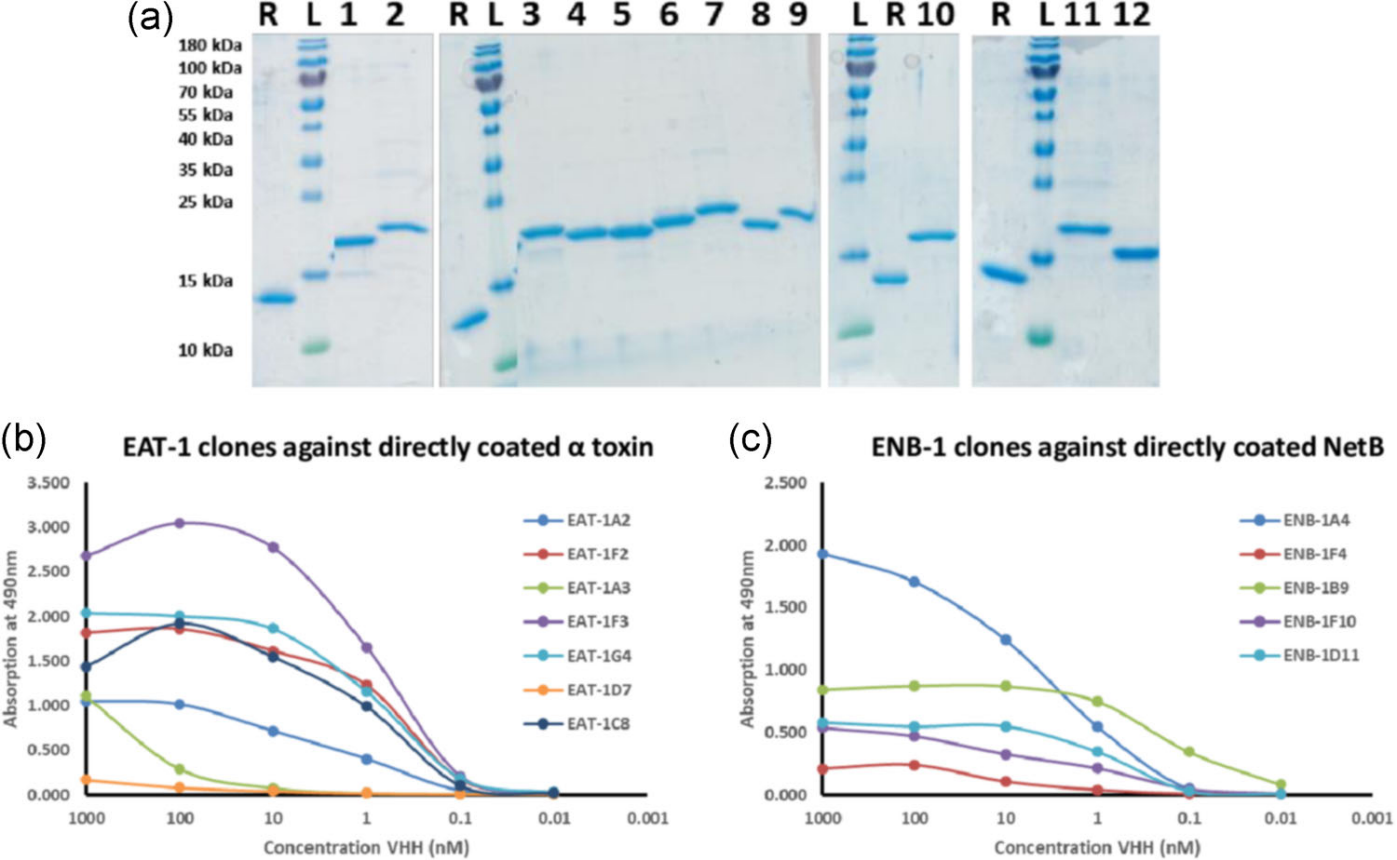
SDS‐PAGE analysis of the purified VHHs and their dose response binding to NetB and α toxin. (a) Coomassie‐stained SDS‐PAGE showing analysis of purified VHH selected on NetB (1–5) and α toxin (6–12). R, 1 µg reference VHH; L, Prestained protein ladder (PageRuler, ThermoFisher); 1, ENB‐1A4; 2, ENB‐1F4; 3, ENB‐1B9; 4, ENB‐1F10; 5, ENB‐1D11; 6, EAT‐1A2; 7, EAT‐1F2; 8, EAT‐1G4; 9, EAT‐1F3; 10, EAT‐1D7; 11, EAT‐1A3; and 12, EAT‐1C8. (b) and (c) Dose response binding of the selected VHHs to α toxin (b) or NetB (c). Representative results are shown from three independent experiments

From the sequenced clones, EAT‐1A2, EAT‐1F2, EAT‐1A3, EAT‐1F3, EAT‐1G4, EAT‐1D7, and EAT‐1C8 from master plate EAT‐1 and ENB‐1A4, ENB‐1F4, ENB‐1B8, ENB‐1B9, ENB‐1F19, and ENB‐1D11 from master plate ENB‐1 were cloned into an expression vector, and VHHs were produced and purified. Table B2 describes the calculation of the VHH concentrations based on absorption (A280) and the correction factor (CF; the extinction factor calculated from VHH sequence) of the VHHs selected on α toxin. Table B3 describes the calculation of the VHH concentrations based on absorption (A280) and the CF of the VHHs selected on NetB. Figure 2a shows SDS‐PAGE analysis of the purity of VHH purified via Immobilized Metal Ion Affinity Chromatography (IMAC). All VHHs appeared to be pure. Clone ENB‐1B8 did not produce any protein.

The purified VHHs were tested for binding to α toxin and NetB to determine the apparent binding affinity. As shown in Figure 2b, very high‐affinity clones were selected, except for EAT‐1A3 and EAT‐1D7, which showed almost no binding. EAT‐1F2, EAT‐1F3, EAT‐1G4, EAT‐1C8, and EAT‐1A2 showed a low nanomolar apparent affinity for α toxin (Figure 2b). For NetB, ENB‐1A4 and ENB‐1F4 had a more moderate affinity, though the Bmax (maximum binding) of ENB‐1F4 was very low (Figure 2c). ENB‐1F10 and ENB‐1D11 had a low nanomolar affinity and ENB‐1B9 had an apparent sub‐nanomolar affinity to the antigen (Figure 2c). Based on these results, the following VHH clones were selected for further testing: EAT‐1A2, EAT‐1F2, EAT‐1F3, EAT‐1G4, and EAT‐1C8 for α toxin and ENB‐1A4, ENB‐1F4, ENB‐1B9, ENB‐1F10, and ENB‐1D11 for NetB.

### 3.2 Selected Nb candidates neutralize NetB and α toxin activity

#### 3.2.1 Neutralization of α toxin activity

**Figure 3:**
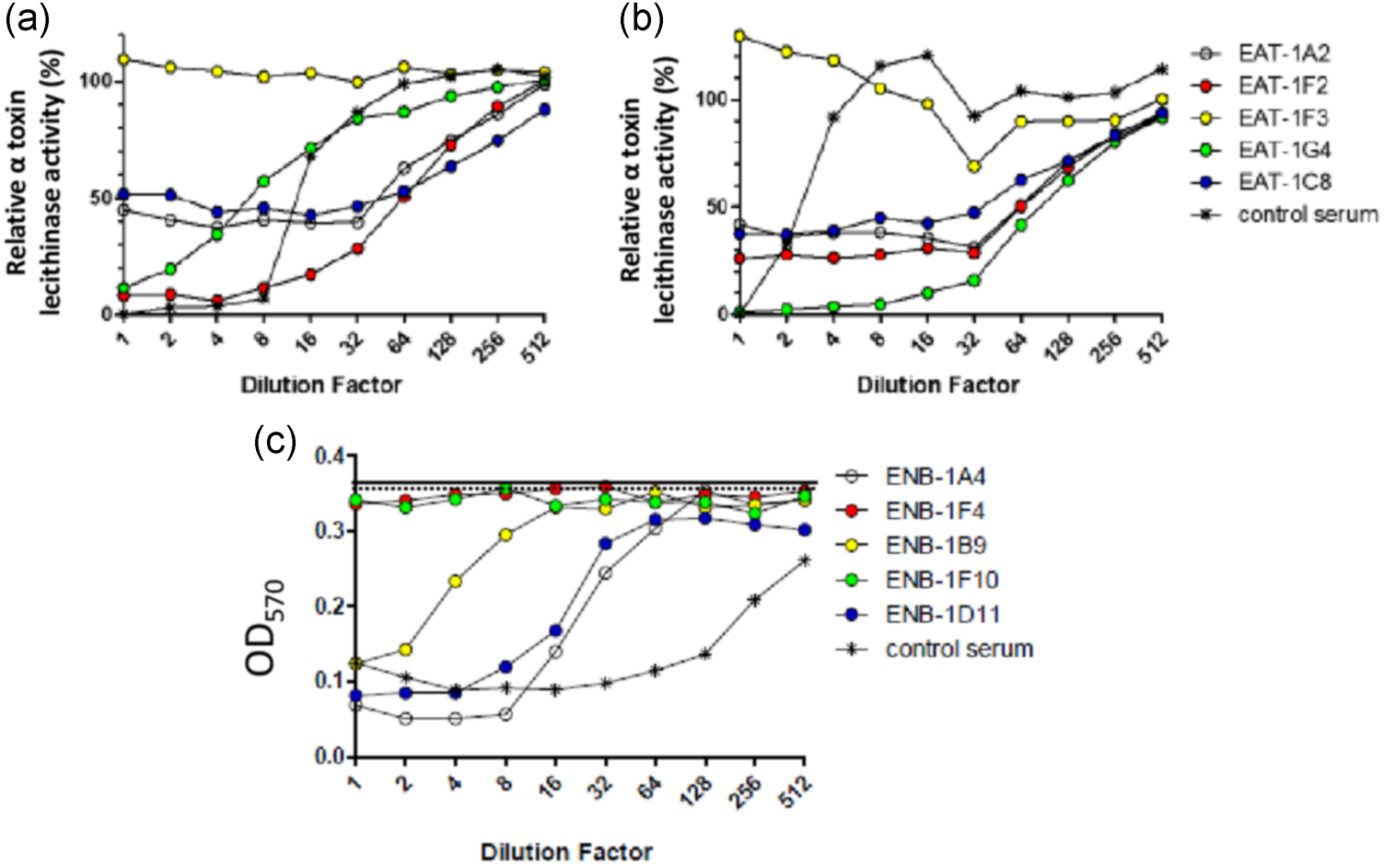
Neutralization of α toxin lecithinase and NetB hemolytic activity by VHH clones. (a, b) A twofold dilution series of VHH antibodies, starting from 5 µM concentration was preincubated with either (a) recombinant α toxin or (b) commercial α toxin, after which egg yolk solution was added. Serum derived from calves immunized with recombinant α toxin was used as a control (control serum). Egg yolk solution incubated with α toxin without VHH, or serum was used to calculate 100% activity. (c) A twofold dilution series of VHH, starting from 5 µM concentration, was preincubated with recombinant NetB (in a total volume of 2 µl), after which 1% chicken erythrocytes was added. Serum derived from rabbits immunized with recombinant NetB was used as a control (control serum). The optical density of 100% hemolysis (mean OD_570_ = 0.37, indicated by solid line) was obtained by diluting the chicken erythrocytes in distilled water. As a control, chicken erythrocytes incubated with NetB, but without VHH or serum was used (mean OD_570_ = 0.36, indicated by dotted line). This resulted in 100% hemolysis. A phosphate‐buffered saline control (1% chicken erythrocytes with no NetB or Nbs) resulted in a mean OD_570_ of 0.03. Representative results are shown from three independent experiments. VHH, Variable domain of the Heavy chain of Heavy chain

The inhibitory capacity of the VHH antibodies towards α toxin lecithinase activity was determined using both commercial and recombinant (used for llama immunization) α toxins. As a control, antisera from calves immunized with recombinant α toxin was used. The control serum was able to neutralize lecithinase activity of both commercial and recombinant α toxins. An eightfold dilution of the antiserum (corresponding to 3.12% serum) was able to completely neutralize the α toxin lecithinase activity of the recombinant α toxin (Figure 3a), whereas only the highest concentration of antiserum (corresponding to 25% serum) was able to completely neutralize lecithinase activity of commercial α toxin (Figure 3b). Considerable difference in inhibitory capacity was seen between the VHH antibodies. VHH EAT‐1F3 did not affect the lecithinase activity of either of the α toxins (Figure 3a,b, yellow). The neutralizing capacity of EAT‐1A2 and EAT‐1C8 was very similar and was the same for both the recombinant and commercial α toxins (Figure 3a,b). The maximal inhibitory capacity was preserved until a 32‐fold dilution (0.16 µM VHH) of the VHHs (Figure 3a,b). However, both EAT‐1A2 and EAT‐1C8 were unable to completely neutralize lecithinase activity, resulting in 40%–50% residual lecithinase activity (Figure 3a,b). The two other VHHs, EAT‐1F2 and EAT‐1G4 showed a difference in neutralizing capacity towards recombinant and commercial α toxins. EAT‐1F2 had a high neutralizing capacity towards recombinant α toxin but was unable to completely neutralize commercial α toxin, resulting in ±25% residual lecithinase activity (Figure 3a,b, red). In contrast to EAT‐1F2, EAT‐1G4 was able to neutralize 100% of the lecithinase activity of the commercial α toxin but was less capable of neutralizing the recombinant α toxin (Figure 3a,b, green).

**Figure A4:**
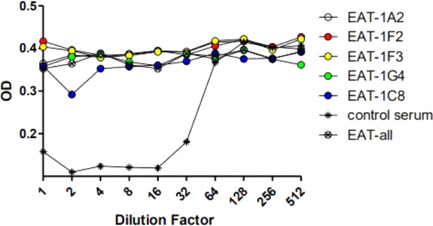
Neutralization of nanobodies α toxin hemolysis by VHH antibodies. A twofold dilution series of VHH antibodies, starting from 5 µM concentration, was preincubated with a commercial α toxin, after which 1% sheep erythrocytes was added. Serum derived from calves immunized with recombinant α toxin was used as a control (control serum, same immunogen as used for llama immunization). The optical density of 100% hemolysis was obtained by diluting the sheep erythrocytes in distilled water. As a control, sheep erythrocytes were incubated with the α toxin, but without VHH antibodies or serum was used. This resulted in 100% hemolysis. The data are representative of three independent experiments. VHH, Variable domain of the Heavy chain of Heavy chain

The inhibitory capacity of the VHH antibodies towards the α toxin hemolytic activity was determined using the commercial α toxin. The recombinant α toxin, which was used to immunize llamas, showed no hemolytic activity. As a control, antisera from calves immunized with recombinant α toxin was used. Up to a 16‐fold dilution of the control serum (corresponding to 1.56% serum) was able to completely inhibit α toxin hemolysis. On the contrary, none of the VHHs affected the hemolytic activity of α toxin (Figure A4). Because the control serum contains polyclonal antibodies and VHHs are monoclonal, the combined effect of all five VHHs towards α toxin was determined (1 µM of each VHH in the highest dilution, corresponding to 5 µM VHHs in total). Combining the VHHs did not affect α toxin hemolysis (Figure A4).

#### 3.2.2 Neutralization of NetB activity

The inhibitory capacity of the VHH antibodies towards NetB activity was determined using the recombinant NetB. The NetB variant W262A, which was used for llama immunization, was not hemolytic. As a control, antisera from rabbits immunized with recombinant NetB was used. The control serum was able to neutralize the hemolytic activity of NetB. VHH antibodies ENB‐1F4 and ENB‐1F10 did not affect NetB hemolysis (Figure 3c, red and green). ENB‐1B9 had intermediate inhibitory capacity, while ENB‐1D11 and ENB‐1A4 were able to neutralize NetB hemolysis up to a four‐ to eightfold dilution (1.25–0.625 µM VHHs) (Figure 3c).

### 3.3 Optimization of candidate VHH clones improves stability and production while retaining affinity

**Figure 4:**
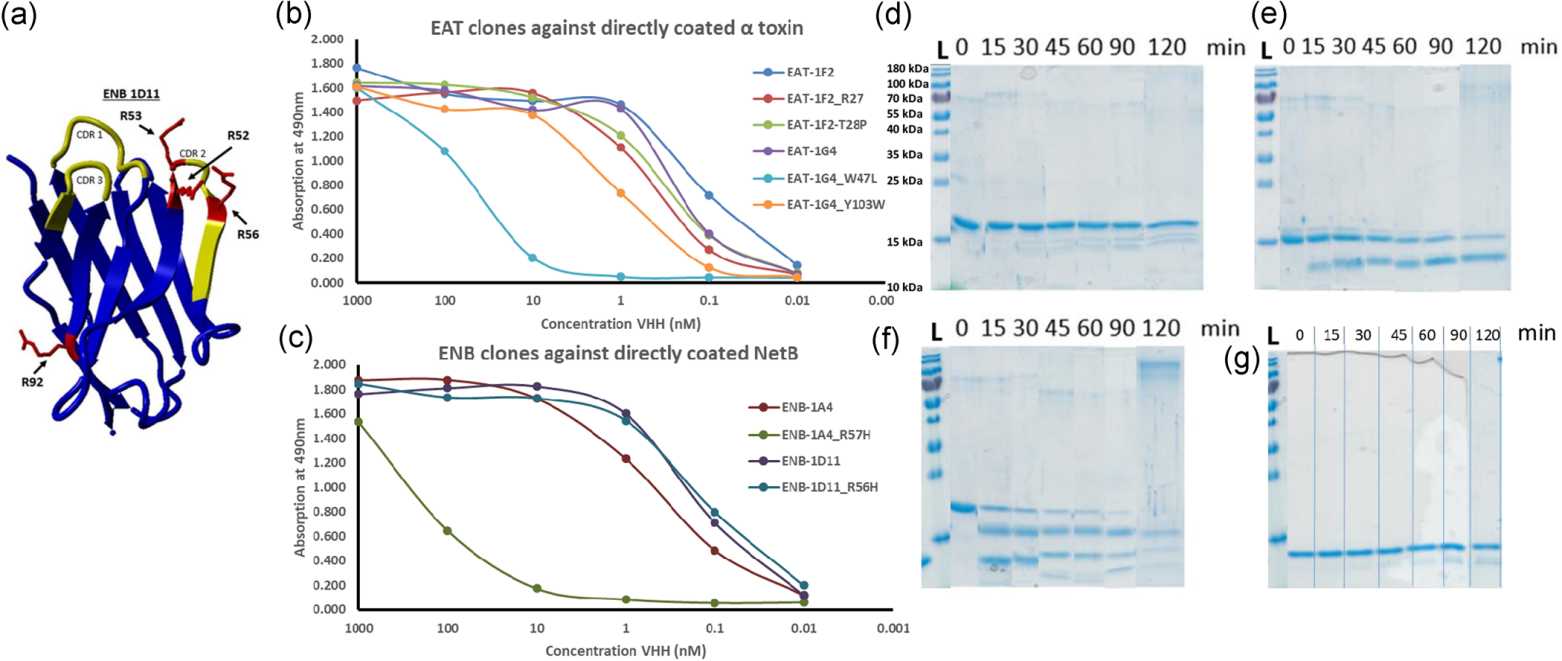
Optimization of VHH clones for improved affinity, production, and stability. (a) Structure of ENB‐ID11 predicted based on homology modeling. The (b, c) Affinity of VHH clones to α toxin (EAT clones) and NetB (ENB clones). (d**–**g) SDS‐PAGE gel of stable VHH control (d), EGFR Q44C (protease susceptible control) (e), EAT‐1F2 (f), and ENB‐1D11_R56H (g) incubated with immobilized trypsin for different time points: 0, 15, 30, 45, 60, 90, and 120 min at 37°C. Representative results are shown from three independent experiments. VHH, Variable domain of the Heavy chain of Heavy chain

The lead clones (EAT‐1G4 and EAT‐1F2 for α toxin; ENB‐1A4 and ENB‐1D11 for NetB) were further optimized for improved affinity, production, and stability using a combination of homology modeling, bioinformatics analysis, and in vitro testing. Figure 4a shows the homology model for ENB‐1D11 (as an example), with the critical amino acids highlighted in red for trypsin susceptibility and CDRs, highlighted in yellow. As shown in Figure 4b, the affinity of IF2 mutants was not affected. While the affinity of 1G4 Y103W was only slightly affected, 1G4 W47L lost affinity (Figure 4b). The affinity of the 1D11 mutant was not affected; however, the 1A4 mutant lost affinity dramatically (Figure 4c). While EAT‐1F2 was susceptible to trypsin (Figure 4f), EAT‐1F2_R27H and EAT‐1F2_T28P clones showed improved trypsin resistance (Table 1). EAT‐1G4 was slightly susceptible to trypsin (Table 1). As EAT‐1G4_W47L and EAT‐1G4_Y103W clones lost affinity, they were not tested for trypsin susceptibility. While ENB‐1A4 was slightly susceptible to trypsin, ENB‐1A4_R57H showed improved trypsin resistance (Table 1). ENB‐1D11 and ENB‐1D11_R56H had similar trypsin resistance (Figure 4g; Table 1). Thermostability testing showed that there were no dramatic changes in the thermostability of all the tested clones (Table 1). Interestingly, the production levels were remarkably increased for most of the mutants (Table 1). Based on these results, EAT‐1G4 and ENB‐1D11_R56H were selected as the lead candidates against α toxin and NetB, respectively, for further engineering into *L. reuteri*.

### 3.4 In silico modeling identifies putative Nb‐interacting epitopes on NetB

**Figure A5:**
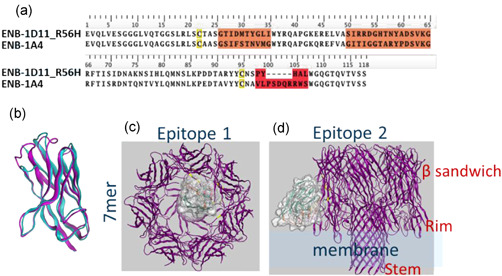
Putative ENB‐ID11_R56H and ENB‐IA4 interacting epitopes on NetB predicted based on in silico structural modeling. (a) Nanobody sequences; the regions of differences are highlighted in brown and red. (b) Homology models of VHH3 (blue) and VHH4 (purple). (c,d) Nanobody interacting epitopes on NetB (β sandwich, rim, and stem domains; shown in red)

NetB is a pore‐forming toxin and active NetB contains seven monomers, which assemble into a ring‐like structure upon contact with cholesterol on the eukaryotic membrane. Each monomer has three domains—β sandwich, rim, and stem; the stem domain is believed to interact with cholesterol. Structural modeling was performed to identify potential Nb interacting epitopes on NetB. As shown in Figure A5, ENB‐1D11_R56H, and ENB‐1A4 seem to interact with epitopes in the rim domain, potentially preventing interaction of NetB with cholesterol and subsequent oligomerization, which is required for NetB toxicity.

### 3.5 *L. reuteri* strains 3630 and 3632 inhibit *C. perfringens* growth

**Figure A6:**
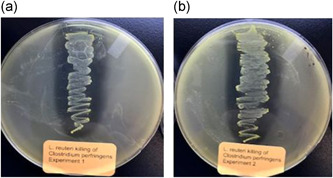
Inhibitory activity of *L. reuteri* 3632 (a) and *L. reuteri* 3630 (b) against *C. perfringens* in an agar overlay assay. The data are representative of three independent experiments

*L. reuteri* 3630 and 3632 vector strains were evaluated for inhibitory activity against *C. perfringens* strain JP1011, a hypervirulent strain isolated from a clinical case of NE and positive for both NetB and α toxin. As shown in Figure A6, both *L. reuteri* strains inhibited *C. perfringens* growth as evident from the clearance zone around the *L. reuteri* streaks.

### 3.6 Global metabolomics analyses of *L. reuteri* 3630 and 3632 culture supernatants identifies potential metabolites with health benefits

An untargeted global metabolomics analysis was performed to identify potential metabolites secreted by *L. reuteri* strains. In total, 433 and 436 known metabolites were detected in the culture supernatants of *L. reuteri* 3630 and 3632, respectively. Of these metabolites, compared to media control, 130 metabolites were secreted 1.5‐fold or higher in the culture supernatant of at least one strain (Table B4). Several tryptophan metabolites such as indolelactate, indolepropionate, indole‐3‐acetamide, indole‐2‐one, and kynurenate were highly enriched in the supernatants of both *L. reuteri* strains compared to media control (Table B4). In addition, other metabolites with potential health benefits were also enriched in the culture supernatants of both *L. reuteri* strains compared to media control and these include alpha‐hydroxyisocaproate, nicotinamide riboside, pantetheine, thymine, daidzein, thioproline, 1‐kestose, alpha‐hydroxyisovalerate, choline phosphate and 2,3‐dihydroxyisovalerate (Table B4).

### 3.7 Global proteomics analyses identify potential native secretion signals and promoters

Global proteomics analyses were performed on *L. reuteri* 3632 cell pellet and supernatant to identify potential native secretion signals and promoters for engineering. This analysis identified 21731 matching spectra, 7263 unique peptides, and 607 proteins in the culture supernatant. Table B5 lists the top 50 proteins with the highest number of matching spectra in the culture supernatant. Similarly, 68,863 matching spectra, 22,676 unique peptides, and 1506 proteins were identified in the cell pellet. Table B6 lists the top 50 proteins with the highest number of matching spectra in the cell pellet. The identified proteins from the culture supernatant were ranked based on the number of matching spectra and seven secretion signals from the highly secreted proteins were selected for further analyses (highlighted in bold in Table B5). Similarly, the proteins from the cell pellet were ranked based on the number of matching spectra, and six promoters from the proteins with the highest number of matching spectra were selected for further testing (highlighted in bold in Table B6).

### 3.8 Assembly of expression cassettes and construction of engineered *L. reuteri* strains

**Figure 5:**
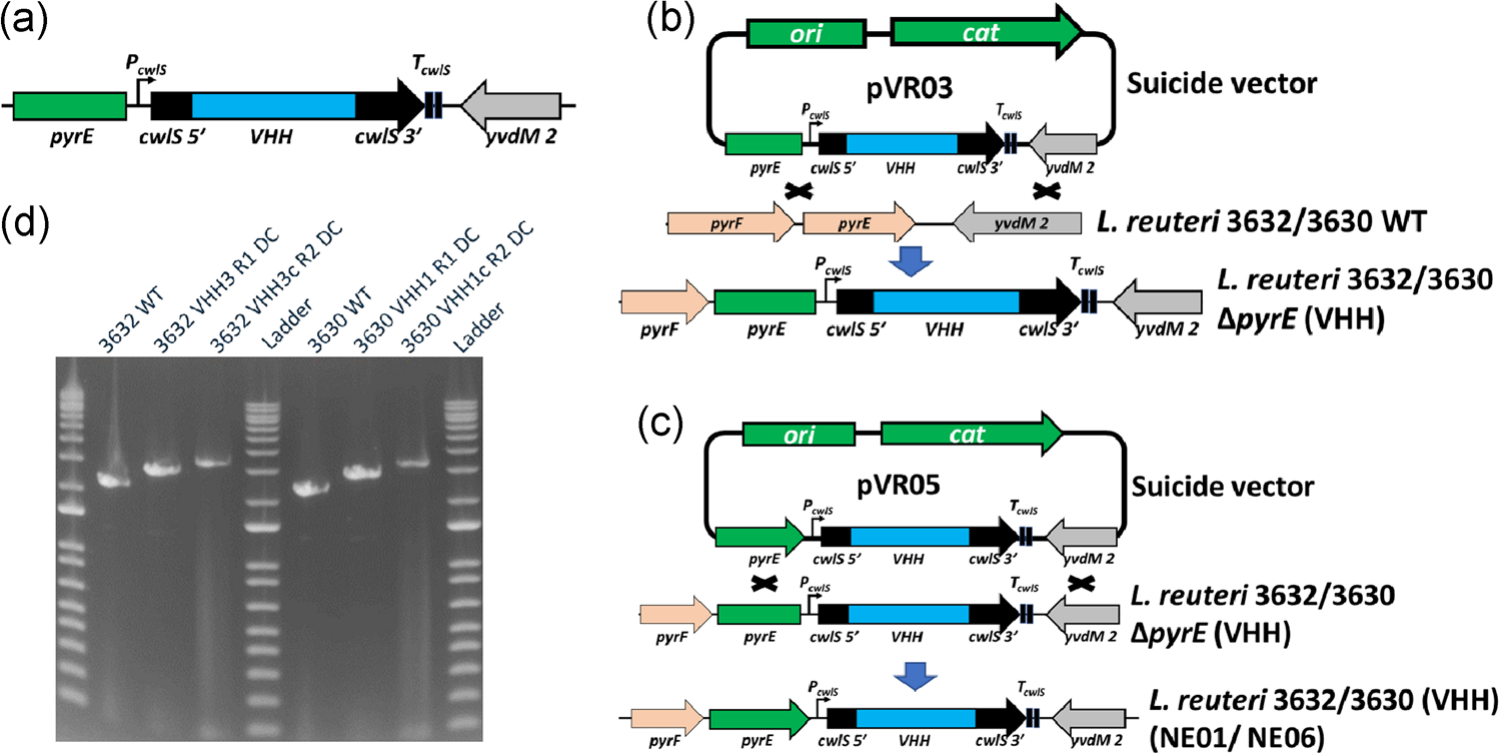
Expression cassette and genetic manipulation toolkit used for integration of expression cassettes into *L. reuteri*genomes. (a) Expression cassette showing the *cwlS* secretion signal sequence, 5′ anchor sequence, optimized ENB‐1D11_R56H (anti‐NetB Nb), *cwlS* 3′ anchor, and *cwlS* terminator and flanking regions. (b) Schematic diagram of the suicide vector used for the integration of expression cassette and *pyrE* truncation (truncated *pyrE* is shown in the solid green block with no arrow), and the integration site in the *L. reuteri* genome. (c) Schematic diagram of the integration vector used for correcting *pyrE* (wildtype *pyrE* is shown in the solid green block with an arrow) and the integration site in the *L. reuteri* genome. (d) Agarose gel showing the PCR confirmation of the integration of the expression cassette and *pyrE* correction for EAT‐1G4 (anti‐α toxin Nb) and ENB‐1D11_R56H (anti‐NetB Nb). 3632 WT, *L. reuteri* 3632; 3632 VHH3 R1 DC, *L. reuteri* 3632 delivering ENB‐1D11_R56H with truncated *pyrE*; 3632 VHH3c R2 DC, *L. reuteri* 3632 delivering ENB‐1D11_R56H with intact *pyrE*; 3630 WT, *L. reuteri* 3630; 3630 VHH1 R1 DC, *L. reuteri* 3630 delivering EAT‐1G4 with truncated *pyrE*; 3630 VHH1c R2 DC, *L. reuteri* 3630 delivering EAT‐1G4 with intact *pyrE*. Representative results are shown from three independent experiments. VHH3, ENB‐1D11_R56H

Based on the preliminary screening of selected promoters and secretion signals, the *cwlS* promoter‐secretion signal combination was selected for assembling the expression cassette. The expression cassette included the native *cwlS* promoter, native *cwlS* secretion signal, optimized VHH sequence, and native *cwlS* terminator (Figure 5a). A suicide vector (integration vector) was generated with the following components: pCG2440 backbone (contains pE194 origin of replication, chloramphenicol resistance marker, and p15A origin of replication; Table 2), 5′ homologous region, expression cassette, and 3′ homologous region (Figure 5a). Initial experiments confirmed that the pCG2440 vector does not replicate in *L. reuteri* 3630 and 3632 and thus is suitable for use as a suicide vector.

**Figure A7:**
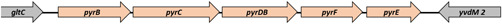
Genomic organization of *pyrE* locus in *L. reuteri* 3630 and 3632 genomes. Note that both *L. reuteri* 3630 and 3632 have identical genomic organization; the genomic organization of only *L. reuteri* 3632 is shown here

To integrate expression cassette into *L. reuteri* genome, we used *pyrE* as a counterselection marker as described previously (Sakaguchi et al., 2013). In *L. reuteri* 3630 and 3632 genomes, *pyrE* (642 bp) is located in an operon with other genes in the order of *pyrB*‐*pyrC*‐*pyrDB*‐*pyrF*‐*pyrE* (Figure A7). We designed integration vectors pVE02 and pVE03 to delete 444 bp of the 3′ coding sequence (CDS) region of *pyrE* and integrate the expression cassette immediately downstream of this deletion (Figure 5b). *L. reuteri* strains with truncated *pyrE* generated from the first round of integration were called NE08 and NE12; these strains deliver Nbs against α toxin (EAT‐1G4) and NetB (ENB‐1D11_R56H), respectively (Figure 5b). In the second round of integration, we generated suicide vectors pVE04 and pVE05 to reconstitute 444 bp of the 3′ CDS of *pyrE* (Figure 5c). The final *L. reuteri* strains with fully integrated expression cassette and reconstituted *pyrE* were called NE01 and NE06; these strains deliver Nbs against α toxin (EAT‐1G4) and NetB (ENB‐1D11_R56H), respectively (Figure 5c). The final strains were confirmed for integration of expression cassette and intactness of *pyrE* by PCR and Sanger sequencing (Figure 5d). As expected, PCR amplification yielded 3200‐bp product for NE01 and NE06, 2800‐bp product for NE08 and NE12, and 2200‐bp product for the parent strains (Figure 5d). NE01 and NE08 were generated using *L. reuteri* 3630 and NE06 and NE12 were generated using *L. reuteri* 3632. We reasoned that using two strains, one delivering NetB‐specific Nb and another delivering α toxin‐specific Nb, allows for efficient production and secretion of Nbs by reducing the burden on the expression and secretion machinery.

### 3.9 Engineered *L. reuteri* strains secrete nbs into the culture supernatant

**Figure 6:**
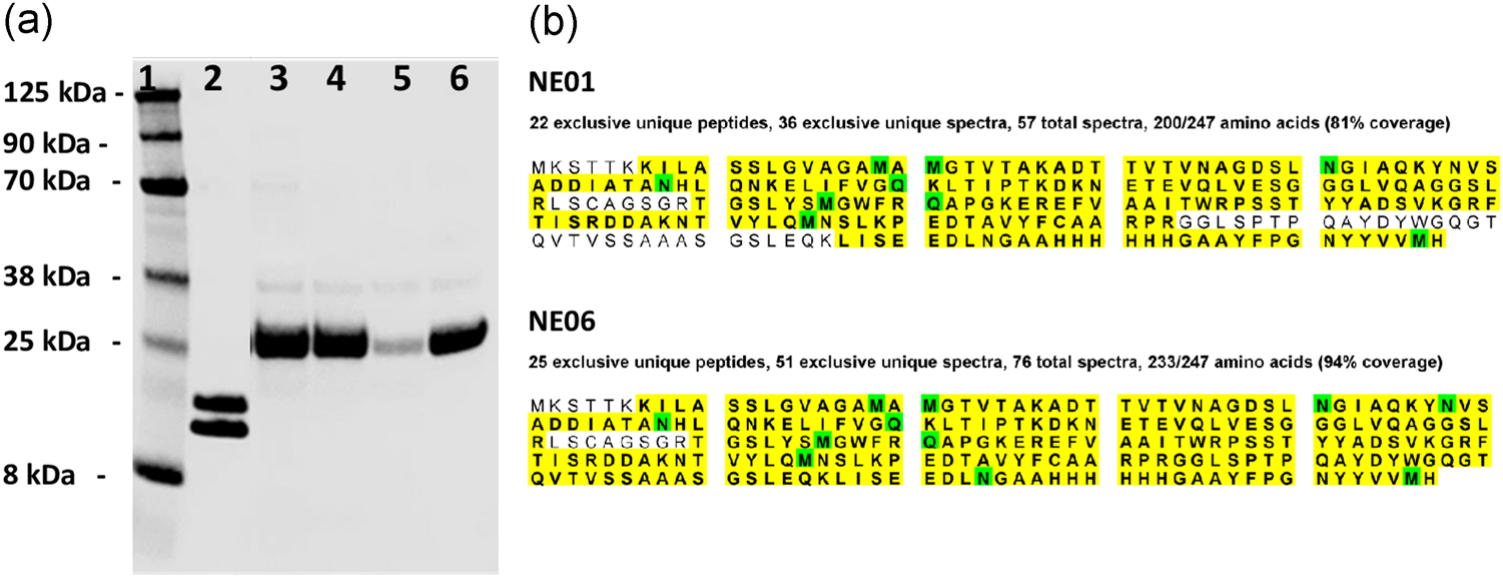
Western blot analysis showing the ammonium precipitated Nbs in the culture supernatant of NE01 and NE06. (a) 1, LiCor protein ladder; 2, ENB‐1D11_R56H control (5 µg, runs as a duplet due to fragmentation); 3, NE01; 4, NE08; 5, NE06; 6, NE12. Please note that the *L. reuteri* secreted Nbs run higher due to the presence of small N‐ and C‐terminal anchors used for efficient secretion. This is a representative blot from three independent experiments. (b) Confirmation of the secreted Nbs in the culture supernatant of NE01 and NE06 by mass spectrometry. The highlighted areas match the nanobody sequence. The residues highlighted in green were identified with a modification (please refer to the methods for details on the modifications included in the database)

The culture supernatants of NE01 and NE06 were evaluated for expression and subsequent secretion of Nbs using western blot. As shown in Figure 6a, expectedly, the anti‐VHH antibody is specifically bound to a protein size of around 25 kDa, which is the expected size of VHHs with anchors. The control VHH was bound to a protein size of around 14 kDa and showed duplet bands (Figure 6a). It should be noted that the *L. reuteri* secreted Nbs run higher due to the presence of small N‐ and C‐terminal anchors from *cwlS* used for efficient secretion. The secreted Nbs were intact, and no degradation was observed for any of the tested strains (Figure 6a). The bands corresponding to Nbs were excised and identified by mass spectrometry, which confirmed that the majority of the spectra matched the respective Nbs (Figure 6b).

### 3.10 Secreted Nbs neutralize NetB activity

**Figure 7:**
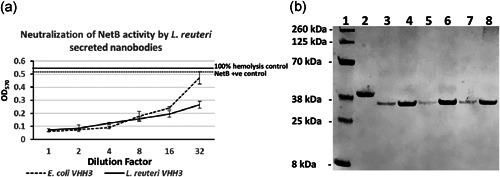
Neutralization of NetB activity by *L. reuteri*secreted Nbs. (a) A twofold dilution series of precipitated VHH antibodies (5 µM) was preincubated with recombinant NetB, after which 1% chicken erythrocytes was added. The optical density of 100% hemolysis was obtained by diluting the chicken erythrocytes in distilled water. As a control, chicken erythrocytes were incubated with NetB, but without Nbs was used. This resulted in 100% hemolysis (OD_570_ = 0.54). A NetB positive control (NetB in PBS) resulted in a mean OD_570_ of 0.52 and a PBS negative control (PBS with no NetB and Nbs) yielded a mean OD_570_ of 0.05. As the initial amounts of *L. reuteri* and *E. coli* purified Nbs used for the assay were different, normalized OD_570_ values are shown. (b) Western blot analysis binding of anti‐NetB Nb to NetB in the culture supernatant from different *C. perfringens* clinical isolates. 1, Ladder; 2, NetB positive control (5 µg); 3, *C. perfringens* JP1011 overnight culture supernatant (10 µl); 4, *C. perfringens* JP1011 overnight culture supernatant, 10× concentrated (10 µl); 5, *C. perfringens* JP1011 mid‐log culture supernatant (10 µl); 6, *C. perfringens* JP1011 midleg culture supernatant, 10× concentrated (10 µl); 7, *C. perfringens* CP1‐1 overnight culture supernatant (10 µl). The data represent the mean ± SD of the results of three independent experiments. Nbs, nanobodies; PBS, phosphate‐buffered saline; VHH, Variable domain of the Heavy chain of Heavy chain

Nbs precipitated from an overnight culture of NE06 were evaluated for their ability to neutralize NetB purified from a clinical isolate of *C. perfringens*. As shown in Figure 7a, the secreted Nbs neutralized hemolytic activity of NetB and this inhibition was maintained up to 32‐fold dilution. The neutralization of NetB activity by Nbs purified from *L. reuteri* was comparable to that of *E. coli* until at least 16‐fold dilution (Figure 7a). The anti‐NetB Nb ENB‐1D11_R56H (VHH3) was also evaluated for its specific binding to NetB from culture supernatants from clinical isolates grown at different growth phases. As shown in Figure 7b, ENB‐ID11_R56H is specifically bound to a band that corresponds to the size of NetB (38 kDa). There was no difference in the amount of NetB produced between mid‐log versus overnight cultures as detected using anti‐NetB Nb (Figure 7b). It should be noted that the NetB positive control runs slightly higher due to the presence of His tag and linker sequence at the N terminus.

### 3.11 Engineered *L. reuteri* strains are genetically and phenotypically stable

**Figure 8:**
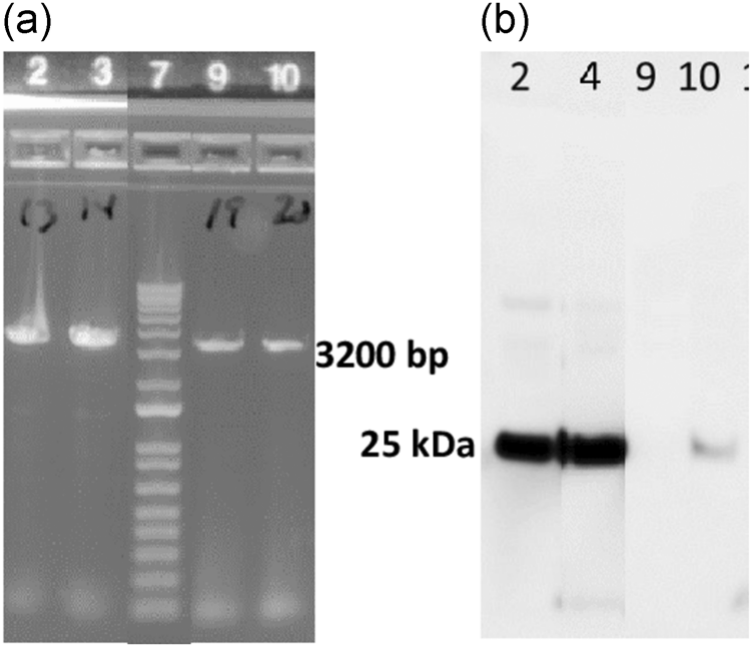
Genomic stability of NE01 and NE06 after 30 passages (approx. 480 generations). (a) PCR confirmation of expression cassette after 30 subsequent passages. Lanes 2 and 3, NE01 passaged 30 times (boxed); 7, eGel 1 kb DNA Ladder; 9 and 10, NE06 passaged 30 times (boxed); all other lanes are not relevant to this study. (b) Western blot confirmation of Nbs secreted by NE01 and NE06 after 30 passages. Lane 2, NE01 passaged 30 times (boxed); lane 4, NE06 passaged 30 times (boxed); lane 9, NE01 whole cell lysate (boxed); lane 10, NE06 whole cell lysate (boxed); all other lanes, not relevant to this study. The results are representative of three independent experiments

The engineered *L. reuteri* strains were tested for their genetic stability after 30 passages (approx. 480 generations). As expected, PCR amplification of the expression cassette using primers that bind to flanking regions outside the expression cassette yielded a product of 3200 bp with NE01 and NE06 (Figure 8a). Sanger sequencing of the expression cassette showed no genetic changes for both strains. Whole‐genome sequencing of NE01 and NE06 confirmed that there were no changes in the expression cassette or flanking regions. Comparative genomics analyses of NE01 and NE06 passaged 30 times with those of 0 passages and their respective parent strains identified no major changes anywhere else in the genomes. Western blot analyses of the culture supernatants of NE01 and NE06 passaged 30 times demonstrated expression and secretion of the respective Nbs similar to those of 0 passages (Figure 8b).

### 3.12 Engineered *L. reuteri* strains show similar colonization levels to that of their parent strains

**Figure 9:**
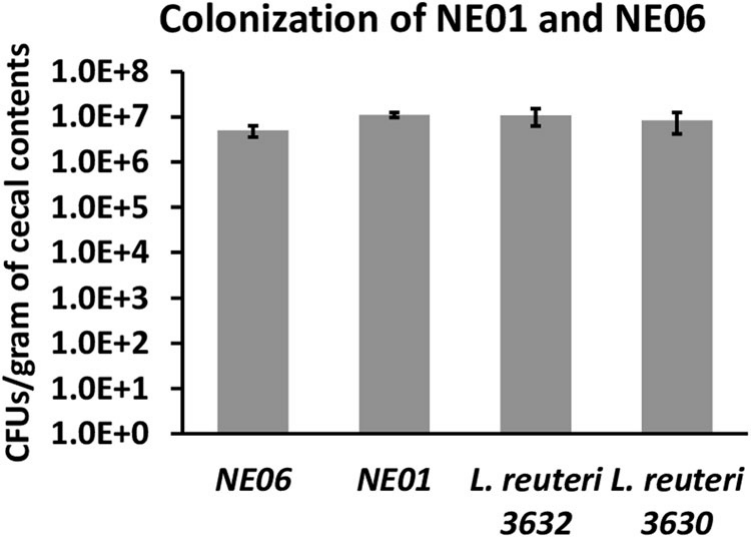
Colonization of NE01 and NE06 in chickens. *L. reuteri* strains were administered via in ovo to 18‐day embryonated chicken eggs and the birds were killed 7 days after hatching and CFUs were quantified from cecal contents. The strains were marked with rifampicin resistance to selectively isolate NE01, NE06, *L. reuteri* 3630, and *L. reuteri* 3632 from the rest of the microbiota from cecal contents. For each group, the data represent the mean ± SD of the results of five chicks

Along with their respective parent strains, NE01 and NE06 were evaluated for colonization in chicken after in ovo administration. As shown in Figure 9, the mean CFUs/g of cecal contents ± SD for *L. reuteri* 3630 and 3632 were 8.36E+06 (±4.14E+06) and 1.07E+07 (±4.49E+06), respectively. Similarly, the mean CFUs/g of cecal contents ± SD for NE01 and NE06 were 1.10E+07 (±1.42E+06) and 4.97E+06 (±1.39E+06) CFUs, respectively (Figure 9). The mean CFUs of all the four strains were found to be not significantly different from each other (*p* > 0.55) (Figure 9). These data suggest that NE01 and NE06 exhibit similar colonization levels to their respective parent strains.

### 3.13 Engineered *L. reuteri* strains partially protect against NE mortality

**Figure 10:**
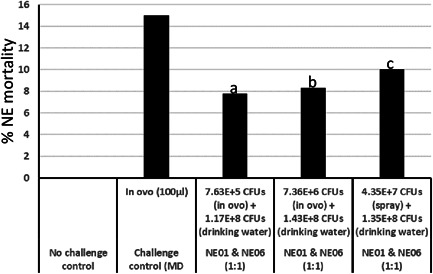
Efficacy of NE01 and NE06 on reduction of NE‐associated mortality. Efficacy of NE01 and NE06 was evaluated using a dual challenge model using *Eimeria maxima* and *C. perfringens* challenge as described in  Section 2. Chickens that died postchallenge phase between 18 (after challenge with *C. perfringens*) and 28 days of age were necropsied, cause of death was listed as NE‐related or non‐NE‐related mortality, and % NE mortality was calculated. ^a^ *p* < 0.05; ^b^ *p* < 0.05; ^c^ *p* = 0.15. CFU, colony‐forming unit; NE, necrotic enteritis

Many strategies have been investigated for experimentally inducing NE; the majority of these strategies use some sort of predisposing factors (Emami & Dalloul, 2021). A dual challenge model that includes the *E. maxima* challenge followed by the *C. perfringens* challenge is a well‐accepted model in the NE field (Emami & Dalloul, 2021). In the present study, a dual challenge model with a primary focus on NE mortality was used for evaluating engineered *L. reuteri* candidates. As shown in Figure 10, while Group 1 (no challenge control) had no NE‐associated mortalities, Group 2 (challenge control) showed 15% NE‐associated mortality (Figure 10). Treatment with NE01 and NE06 administered via in ovo at 7.63 × 10^5^ CFUs/dose and drinking water at 1.17 × 10^8^ CFUs/dose (Group 3) significantly reduced NE‐associated mortality to 7.8% (Figure 10; *p* < 0.05 compared to Group 2). Treatment with NE01 and NE06 administered via in ovo at 7.36 × 10^6^ CFUs/dose and drinking water at 1.43 × 10^8^ CFUs/dose (Group 4) significantly reduced NE‐associated mortality to 8.33% (Figure 10; *p* < 0.05 compared to Group 2). Treatment with NE01 and NE06 administered via spray at 4.35 × 10^7^ CFUs/dose and drinking water at 1.35 × 10^8^ CFUs/dose (Group 5) numerically reduced NE‐associated mortality to 10.0% (Figure 10; *p* = 0.15 compared to Group 2). Preventative fraction is a ratio used in epidemiological studies to assess the impact of vaccination on a disease. The preventative fractions for Group 3, Group 4, and Group 5 were 48%, 44%, and 33%, respectively (Figure 10). These data suggest that a two‐dose administration of NE01 and NE06 partially protects chickens from NE.

## 4 DISCUSSION

In this study, we developed *L. reuteri* as a live vector for in situ delivery of Nbs against NetB and α toxin from *C. perfringens*. We generated several Nb candidates that successfully neutralized NetB and α toxin from phage display libraries derived from immunized llamas. We showed that vector strains *L. reuteri* 3630 and 3632 inhibit *C. perfringens* in vitro, and secrete over 130 metabolites, some of which have been shown to play a key role in maintaining intestinal integrity, regulating microbiota, and reducing inflammation. We also demonstrated that engineered *L. reuteri* strains can efficiently produce and secrete Nbs and that these Nbs neutralize NetB. The engineered *L. reuteri* strains were genetically and phenotypically stable and showed persistent colonization in vivo similar to their parent strains. More importantly, a two‐dose in ovo and drinking water administration of engineered *L. reuteri* candidates reduced NE mortality in chickens.

**Figure 11:**
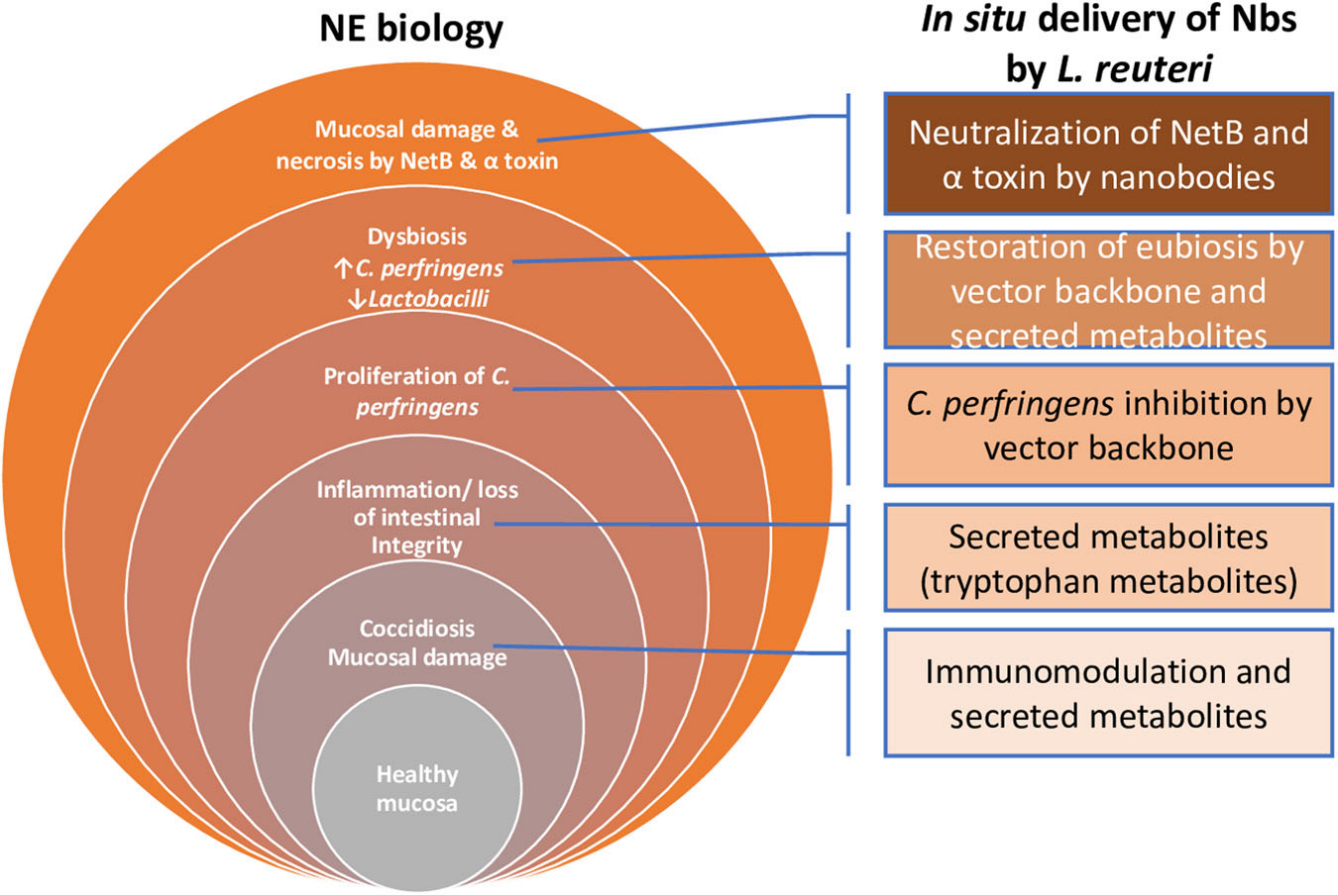
Schematic model showing the possible mechanisms of action of *L. reuteri* vector strains and secreted Nbs to address different aspects of NE biology. Nbs, nanobodies; NE, necrotic enteritis

NE is a complex disease, and successful control of NE requires a multifactorial approach (Williams, 2005). *C. perfringens* is the primary causative agent of NE (Parish, 1961). Several factors such as *Eimeria* infection, dietary factors, immunosuppression, and *Fusarium* mycotoxins have been identified to predispose birds to NE (Williams, 2005). These factors cause damage to the intestinal layer and facilitate *C. perfringens* proliferation and toxin production. Growth of *C. perfringens* results in drastic shifts in microbiota with a reciprocal decrease in Lactobacilli (Yang, Liu, Robinson, et al., 2021; Yang, Liu,  Wang, et al., 2021). We selected *L. reuteri* strains with intrinsic benefits as delivery vectors. More specifically, *L. reuteri* 3630 and 3632 inhibited the growth of pathogenic isolates of *C. perfringens* in vitro, suggesting that the backbones have the potential to inhibit *C. perfringens* proliferation in vivo and prevent resulting dysbiosis. Using global metabolomics analysis, we showed that *L. reuteri* 3630 and 3632 secrete several metabolites with potential microbiota modulation and anti‐inflammatory activities. Tryptophan metabolites were among one of the highly secreted metabolites by *L. reuteri* 3630 and 3632; tryptophan metabolites have been previously shown to play a key role in regulating the immune system and local microbiota, maintaining intestinal integrity, and reducing inflammation, most likely via activation of aryl hydrocarbon receptor pathway (Galligan, 2018; Gao et al., 2018; Negatu et al., 2020; Scott et al., 2020). These data suggest that vector backbones potentially work synergistically with Nbs to address other aspects of NE biology such as *C. perfringens*, dysbiosis, and inflammation (Figure 11).

NetB and α toxin, produced by *C. perfringens*, are believed to be the main toxins involved in the pathogenesis of NE (Keyburn et al., 2008; Kiu et al., 2019). Alpha toxin is the most toxic enzyme produced by *C. perfringens* type A strains and hydrolyzes two major constituents of the eukaryotic membrane (phosphatidylcholine [lecithin] and sphingomyelin) causing membrane disruption and cell lysis (Nagahama et al., 1998; Urbina et al., 2009). We developed several Nbs that neutralize α toxin activity. Different Nbs had different inhibitory capacities towards the lecithinase activity of recombinant and commercial α toxins. This differential inhibitory capacity of the VHHs might have different explanations. First, the recombinant α toxin has a C‐terminal His tag, whereas the commercial α toxin is purified from *C. perfringens* and has no tags. Although an effect of the His tag is unlikely, it cannot be excluded. Next, it should be noted that the recombinant α toxin is derived from an intestinal isolate, whereas the origin of the commercial α toxin is unknown. A difference in α toxin derived from enteric *C. perfringens* isolates and gas gangrene isolates has been previously reported, with higher trypsin resistance for α toxin of the enteric isolates (Uzal et al., 2014). As the origin of the commercial α toxin is unknown, it cannot be excluded that it has a slightly different activity.

NetB is a heptameric beta pore‐forming toxin that forms single channels in planar phospholipid bilayers (Savva et al., 2013). NetB activity is primarily influenced by cholesterol, which enhances the oligomerization of NetB and plays an important role in pore formation (Savva et al., 2013). NetB has high hemolytic activity towards avian red blood cells (Yan et al., 2013). In the present study, we developed several Nbs that neutralize the hemolytic activity of NetB. The protective range for the top two Nbs ENB‐1D11 and ENB‐1A4 for neutralizing 20 µg of NetB ranged from 5 µg to 0.312 ng. The polyclonal antisera generated against recombinant NetB in rabbits showed a broader protection range of 25% serum to 0.019% serum. The differential inhibitory activity of VHH and polyclonal antisera is likely due to the presence of multiple neutralizing antibodies in the antisera compared to the monoclonal nature of VHHs. NetB epitope mapping by in silico analysis showed that ENB‐1D11_R56H and ENB‐1A4 seem to interact with epitopes in the rim domain and prevent interaction with cholesterol and subsequent oligomerization, which is key for NetB toxic activity (35).

The *pyrE* gene was used as a counterselection marker to engineer *L. reuteri* strains to deliver Nbs against NetB and α toxin (Sakaguchi et al., 2013). *pyrE* encodes for orotate phosphoribosyl transferase, which converts orotic acid to orotidine 5′‐monophosphate (OMP). PyrE also metabolizes 5‐FOA, an analog of orotic acid, into 5‐fluoroorotidine monophosphate (5‐FOMP); the accumulation of 5‐FOMP is toxic and leads to cell death. Double crossover integrants were selected from single crossover integrants by plating on MRS containing 5‐FOA and uracil. 5‐FOA is toxic to single crossover integrants as they still contain intact *pyrE*, whereas double crossover integrants grow on 5‐FOA. In addition, *pyrE* deletion makes the strain uracil auxotroph; the truncated *pyrE* mutants were unable to grow in the absence of exogenous uracil. Given that *pyrE* truncated strains are uracil auxotrophs, *pyrE* truncation can also serve as a safety and biological containment strategy to reduce the survival of engineered strains in the environment (Heap et al., 2014). It should be noted that all the final engineered *L. reuteri* strains generated using PyrE counterselection contained no antibiotic resistance markers.

Western blot analysis confirmed that the engineered strains were able to secrete Nbs into the culture supernatant. Mass spectrometry analysis further confirmed that the bands detected in western blot indeed correspond to secreted Nbs. Western blot analysis of the bacterial cell pellet (Figure 8) and the culture supernatant also showed that the majority of the Nbs are secreted into the culture supernatant and only a small portion of the Nbs are located intracellularly and/or on the cell surface. Protein quantification estimated that the total amount of Nbs secreted in the supernatant totaled around 5 mg/L. Functional analysis showed that the secreted Nbs neutralized NetB activity. Western blot analysis of the culture supernatant from clinical isolates of *C. perfringens* showed that NetB‐specific Nb specifically binds to a protein around 30 kDa, which corresponds to the size of NetB. These data suggest that the engineered strains can efficiently secrete Nbs and that these Nbs are functional.

Genetic and phenotypic stability is of paramount importance for developing a microbial strain as an in situ delivery vector. Using PCR and Sanger sequencing, we showed that our engineered *L. reuteri* strains had intact expression cassette and flanking regions, with no mutations, even after 30 passages (approx. 480 generations) for 30 days. Whole‐genome sequencing not only confirmed the integrity and the intactness of the expression cassette and the flanking regions but also showed that there were no major mutations anywhere else in the genome. Furthermore, western blot analysis showed that *L. reuteri* passaged for 30 times expressed and secreted Nbs similar to those of 0 passage. The data suggest that the engineered strains are genetically and phenotypically stable.

Safety is of utmost importance in developing MVs for the delivery of biomolecules. Species belonging to *Lactobacillus* have been used in fermented foods for decades (Bourdichon et al., 2012). Lactobacilli are also a normal part of the GI microbiota of all vertebrates, including humans, monkeys, chickens, turkeys, doves, pigs, dogs, lambs, cattle, and rodents (Valeur et al., 2004). Several *L. reuteri* strains have been notified to FDA as “Generally Regarded As Safe” for use in specific foods or as new dietary ingredients (Gangaiah et al., 2021). In addition, *L. reuteri* species are also considered safe for use in, or as a source of food for, human and animal consumption by EFSA, which has granted QPS status for *L. reuteri* species (Hazards et al., 2020). Several clinical studies have been conducted with *L. reuteri* species in humans and other species, including immunocompromised patients with no or very few adverse events (Indrio et al., 2008; Mu et al., 2018; Wolf et al., 1998). Previous in silico, in vitro, and in vivo analyses in chickens showed that the vector strains *L. reuteri* 3630 and 3632 have a favorable safety profile (Gangaiah et al., 2021). A 28‐day subchronic toxicity study showed that rats can tolerate high doses of *L. reuteri* 3630 and 3632 and no adverse events were observed when male and female Sprague Dawley rats were administered with 1.6E+10 CFUs of *L. reuteri* 3630/kg body weight/day plus 5.7E+10 CFUs of *L. reuteri* 3632/kg body weight/day for 28 days. The backbone strain *L. reuteri* 3632 has also been tested via feed administration for 50 days at 1 × 10^7^ CFUs/g of feed in swine with no adverse events. No adverse effects on hatchability or on chicks after hatching were observed in chickens when administered in ovo to 18‐day old embryos at a dose of 1 × 10^5^, 1 × 10^6^, or 1 × 10^7^ CFUs/embryo (hatchability between 80% and 85%—all groups had similar hatchability). A two‐day spray (Day 0, 1 × 10^7^ CFUs/bird) and drinking water (Day 13, 1 × 10^8^ CFUs/bird) administration to chickens also showed no adverse events. Drinking water administration of the backbone strain *L. reuteri* 3632 at 1 × 10^8^ CFUs/bird/day every day for 21 days showed no toxicity or adverse events (Table C1). Together, these data suggest that the vector strains have a favorable safety profile and are suitable as delivery vectors.

In vivo efficacy depends on several factors, including colonization and persistence of the vector strains and expression and stability of the secreted Nbs in the gut. Following single in ovo administration, both vector strains showed persistent colonization and this colonization remained unchanged until Day 7 after hatching (study end). In a follow‐up study, two‐dose administration of NE01 and NE06 on Day 0 via spray and on Day 13 via drinking water resulted in persistent colonization of strains until at least 28 days after the spray administration with approximately 5 × 10^3^ CFUs/g of cecal contents recovered on Day 28, suggesting that the strains remain colonized for the entire duration of NE phase. In addition, the promoter used for delivering Nbs comes from the *cwlS* gene, which is an essential gene involved in cell separation, a key process that concludes the process of cell division (Fukushima et al., 2006); thus, it is highly likely that this promoter will be active in vivo during replication and drive the production of Nbs. Trypsin is one of the primary proteolytic enzymes in the small intestine, which has the potential to degrade *L. reuteri* delivered Nbs; however, our in vitro trypsin digestion data showed that our lead Nb candidates are resistant to supraphysiological levels of trypsin and hence can withstand physiological levels of trypsin present in the small intestine. A small intestinal environment has a pH of around 5.7–6.5 (M. Mabelebele et al., 2002); we have shown that Nbs purified from overnight *L. reuteri* cultures, which consistently reach a final pH of 3.5‐4.5, remain active and functional, suggesting that our Nbs can withstand exposure to low pH.

In nature, NE occurs in two forms—acute clinical form and chronic subclinical form (Emami & Dalloul, 2021; Emami et al., 2021; Hofacre et al., 2018). Clinical NE is characterized by extensive necrosis of the small intestinal mucosa and high mortality (Emami & Dalloul, 2021; Emami et al., 2021; Hofacre et al., 2018). Subclinical NE is characterized by damage to the intestinal mucosa resulting in decreased digestion and absorption, reduced weight gain, and increased feed conversion ratio (Emami & Dalloul, 2021; Emami et al., 2021; Hofacre et al., 2018). While clinical NE models use mortality as the primary study parameter, subclinical models use lesion score as the primary study parameter. In this study, we showed that oral administration of NE01 and NE06 delivering Nbs via in ovo/spray and drinking water partially reduces NE mortality. We have also shown that administration of NE01 and NE06 in drinking water every day for 21 days reduces both NE mortality and lesion score (Table C1). Given that our strains can reduce lesion score, one might rationalize that administration of NE01 and NE06 also leads to secondary benefits in terms of improved weight gain and feed conversion ratio (FCR). A two‐dose administration of NE01 and NE06 via in ovo and drinking water showed marginal, numerical improvement in FCR; however, none of these comparisons were statistically significant compared to challenge control (Group 2) (Groups 1, 2, 3, 4, and 5 had a mean adjusted FCR of 1.65, 2.06, 1.98, 1.96, and 1.95, respectively). A larger study with a greater number of birds is likely required to observe the significant effect of two‐dose administration of NE01 and NE06 on performance. Alternatively, a two‐dose administration of these candidates may not be sufficient to have a significant effect on performance.

**Figure C1:**
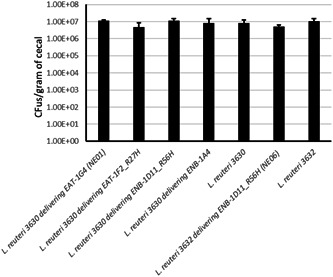
Colonization of engineered *L. reuteri* candidates. *L. reuteri* strains were administered via in ovo to 18‐day embryonated chicken eggs and the birds were killed 7 days after hatching and CFUs were quantified from cecal contents. The strains were marked with rifampicin resistance to selectively isolate NE01, NE06, *L. reuteri* 3630, and *L. reuteri* 3632 from the rest of the microbiota from cecal contents. For each group, the data represent the mean ± SD of the results of five chicks

One might wonder that the observed in vivo efficacy might be solely due to the general antimicrobial and/or probiotic effect of the vector backbones. However, as shown in Table C1, administration of *L. reuteri* 3630 delivering EAT‐1G4 (NE01, Nb against α toxin), *L. reuteri* 3630 delivering EAT‐1F2_R27H (Nb against α toxin), and *L. reuteri* 3630 delivering ENB‐1A4 (Nb against NetB) separately in drinking water reduced NE‐associated mortality by 62.6%, 27.2%, and 16.0% and NE lesion score by 72.2%, 47.2%, and 19.4%, respectively. Of the three strains, only NE01 showed a statistically significant reduction in NE‐associated mortality and lesion score; the other two strains showed a numerically marginal reduction in mortality and lesion score. All the above three strains expressed similar levels of Nbs in vitro and colonized chicken gut to a similar level (Figure C1). Thus, the *L. reuteri* 3630 backbone delivering three different Nb clones separately had a similar ability to express and secrete Nbs and colonize chicken gut but showed variable efficacy, suggesting that the *L. reuteri* 3630 vector backbone contributes minimally to the observed efficacy and that most of the efficacy is contributed by Nbs.

Administration of *L. reuteri* 3630 delivering ENB‐1D11_R56H (Nb against NetB) and *L. reuteri* 3632 delivering ENB‐1D11_R56H (NE06) together in drinking water significantly reduced NE‐associated mortality by 44% and lesion score by 61.1% (Table C1); the two strains also expressed and secreted similar levels of Nbs in vitro and showed similar colonization as the other engineered strains and their parent backbones (Figure C1). Given that *L. reuteri* 3630 vector backbone contributes minimally to the efficacy, one might hypothesize that the entire efficacy of the combination strains might be contributed by *L. reuteri* 3632 backbone via antimicrobial effect against *C. perfringens*. Both, *L. reuteri* 3630 and *L. reuteri* 3632 had similar inhibitory activity against *C. perfringens*; however, *L. reuteri* 3630 contributed minimally to in vivo efficacy. This suggests that *L. reuteri* 3632 backbone also contributes minimally to the observed efficacy of the combination strains. As NetB is required for induction of NE, it is highly likely that neutralization of NetB by *L. reuteri* secreted Nbs contribute to most of the total efficacy observed with combination strains.

In conclusion, the results presented in this study demonstrate the potential of using *L. reuteri* as a live vector for the delivery of Nbs to reduce NE in poultry. The *L. reuteri* vectors are designed and developed as modular platforms, and have a favorable safety profile in chickens, pigs, and rats; the data presented in this study open new avenues for exploring these strains for other disease indications across animal and human health. The dual effects from the vector backbone and Nbs are key to addressing complex diseases like NE; the potential of the platform to address other similar complex diseases is also worth exploring. A better understanding of the factors that influence the dose of the delivered target in vivo is key to successful and reproducible efficacy; future studies will focus on a deeper understanding of the colonization and survival dynamics of the vector strains as well as expression, secretion, and stability of the target molecules in vivo at the site of action. Future investigations will also focus on a better understanding of PyrE as a biological containment strategy to reduce or prevent the environmental dissemination of vector strains.

## CONFLICTS OF INTEREST

Dharanesh Gangaiah, Valerie Ryan, Shrinivasrao P. Mane, Enid T. McKinley, Nandakumar D. Reddy, and Arvind Kumar are employees of Elanco Animal Health Inc. Nallakannu Lakshmanan was an employee of Elanco Animal Health Inc. at the time the work was done. Elanco Animal Health Inc. is a company that develops, manufactures, and sells veterinary pharmaceuticals and nutritionals. Daphne van Hoese and Edward Dolk are current employees of QVQ Holding BV, which discovers and develops nanobodies for different target diseases.

## ETHICS STATEMENT

All chickens were housed and cared for under the Guide for the Care and Use of Agricultural Animals in Research and Teaching and all local standard operating procedures. The study was reviewed and approved by the Animal Care and Use Committee of the institution performing the study (Assigned ACUP #1622).

## AUTHOR CONTRIBUTIONS

**Dharanesh Gangaiah**: conceptualization (equal); investigation (lead); methodology (lead); visualization (lead); formal analysis (lead); supervision (equal); writing—original draft (lead); writing—review and editing (equal). **Valerie Ryan**: conceptualization (equal); investigation (equal); methodology (equal); visualization (equal); writing—review and editing (equal). **Daphne van Hoesel**: investigation (equal); methodology (equal); writing—review and editing (equal). **Shrinivasrao P. Mane**: investigation (equal); methodology (equal); writing—review and editing (equal). **Enid T. McKinley**: investigation (equal); methodology (equal); writing—review and editing (equal). **Nallakannu Lakshmanan**: investigation (equal); methodology (equal); Writing— review and editing (equal). **Nandakumar D. Reddy**: investigation (equal); methodology (equal); writing—review and editing (equal). **Edward Dolk**: investigation (equal); methodology (equal); writing—review and editing (equal). **Arvind Kumar**: conceptualization (equal); funding acquisition (lead); investigation (equal); project administration (lead); resources (lead); supervision (equal); writing—review and editing (equal).

## Data Availability

All data generated or analyzed during this study are included in this published article.

## APPENDIX A

See Figures A1, A2, A3, A4, A5, A6, and A7.

## APPENDIX B

See Tables B1, B2, B3, B4, B5, and B6.

**Table B1:** The size and measured OD_600_ of the cDNA libraries

Llama	Library size	OD_600_
SNL‐133, Day 43	9.6 × 10^8^	78.3
SNL‐134, Day 43	6.4 × 10^8^	73.4
SNL‐133, Day 78	9.6 × 10^7^	49.3
SNL‐134, Day 78	4.8 × 10^7^	36.8

Abbreviation: cDNA, complementary DNA.

**Table B2:** Calculation of the VHH concentration based on absorbance (*A*_280_) and the correction factor (CF, the extinction factor calculated from VHH sequence) of the VHH selected on α toxin

VHH	Vector	Tag	Correction factor	Molecular weight	*A*_280_	Concentration (mg/ml)
EAT‐1A2	pMEK222	FLAG‐His	1.795	15,906	2.506	1.396
EAT‐1F2	pMEK222	FLAG‐His	1.802	15,843	0.732	0.406
EAT‐1A3	pMEK222	FLAG‐His	1.706	16,127	0.766	0.449
EAT‐1F3	pMEK222	FLAG‐His	2.193	15,525	1.996	0.910
EAT‐1G4	pMEK222	FLAG‐His	1.401	14,324	0.826	0.590
EAT‐1D7	pMEK222	FLAG‐His	1.855	15,387	1.074	0.579
EAT‐1C8	pMEK222	FLAG‐His	1.626	15,721	1.618	0.995

**Table B3:** Calculation of the VHH concentration based on absorbance (*A*_280_) and the correction factor (CF, the extinction factor calculated from VHH sequence) of the VHH selected on NetB

VHH	Vector	Tag	Correction factor	Molecular weight	*A*_280_	Concentration (mg/ml)
ENB‐1A4	pMEK222	FLAG‐His	1.679	15,227	1.950	1.161
ENB‐1F4	pMEK222	FLAG‐His	1.303	16,547	0.336	0.258
ENB‐1B8	pMEK222	FLAG‐His	1.279	15,686	0.000	‐
ENB‐1B9	pMEK222	FLAG‐His	1.788	15,128	3.598	2.012
ENB‐1F10	pMEK222	FLAG‐His	1.448	14,886	0.676	0.467
ENB‐1D11	pMEK222	FLAG‐His	1.455	14,817	2.230	1.533

Abbreviations: ‐, data not available; VHH, variable domain of the heavy chain of the heavy chain.

**Table B4:** Top 130 metabolites secreted by *L. reuteri* 3630 and 3632 into culture supernatant

Metabolite^a^	*L. reuteri* 3630^b^	*L. reuteri* 3632^b^
Tricarballylate	113.46	64.47
**Indolelactate**	**98.94**	**99.86**
**Alpha‐hydroxyisocaproate**	**83.22**	**73.14**
Mannitol/sorbitol	75.20	68.08
Mevalonolactone	71.05	37.22
3‐(4‐Hydroxyphenyl)lactate	62.79	50.22
Phenyllactate (PLA)	50.46	48.57
Dihydroorotate	44.47	31.30
Orotidine	41.03	34.38
Gamma‐glutamylisoleucine	35.72	34.17
*N*‐delta‐acetylornithine	31.05	27.31
Mevalonate	29.67	19.80
2‐Keto‐3‐deoxy‐gluconate	25.91	25.57
*N*‐acetylglucosamine/*N*‐acetylgalactosamine	22.88	14.50
Syringic acid	17.24	19.10
Lactate	17.17	16.26
**Nicotinamide riboside**	**16.61**	**16.73**
Orotate	14.72	13.47
*N*‐acetylserine	14.25	12.88
*N*‐formylmethionine	13.52	11.86
2‐Hydroxy‐3‐methylvalerate	13.02	8.58
*N*‐acetylthreonine	11.87	9.71
*N*‐acetylaspartate (NAA)	11.00	8.72
N‐acetylmethionine	10.22	7.81
Glucose 6‐phosphate	10.21	12.94
*N*‐carbamoylaspartate	9.95	9.72
Succinate	9.59	12.13
Ornithine	7.10	7.18
(3′–5′)‐adenylylguanosine	6.53	3.37
**Pantetheine**	**5.84**	**1.68**
*N*‐acetylglutamate	5.81	4.71
**Thymine**	**5.51**	**5.22**
Galactonate	5.01	5.35
Citrulline	4.82	6.21
**Daidzein**	**4.52**	**3.19**
*N*‐acetylmethionine sulfoxide	4.37	4.20
(3′–5′)‐cytidylyluridine	4.31	2.97
*N*6,*N*6‐dimethyllysine	4.27	5.06
Pyrraline	4.10	4.20
Sebacate (C10‐DC)	4.04	3.92
2‐Oxoarginine	3.83	4.30
5‐Methylcytosine	3.63	3.19
Cysteine sulfinic acid	3.58	3.77
Ribulose/xylulose	3.55	2.74
**Indolepropionate**	**3.47**	**3.91**
Methionine	3.43	4.39
Glutamine	3.35	10.01
Beta‐citrylglutamate	3.35	3.48
*N*‐acetyl‐cadaverine	3.29	4.09
Pyruvate	3.25	6.71
2‐Hydroxy‐4‐(methylthio)butanoic acid	3.23	3.37
Inositol 1‐phosphate (I1P)	3.23	2.18
3,4‐Dihydroxybutyrate	3.06	2.84
4‐Hydroxy‐2‐oxoglutaric acid	2.98	1.88
3‐Hydroxydecanoate	2.97	2.65
(3′–5′)‐adenylyluridine	2.95	2.01
Adenine	2.94	3.79
4‐Hydroxyglutamate	2.94	2.87
2‐Hydroxybutyrate/2‐hydroxyisobutyrate	2.84	2.43
*N*‐acetylasparagine	2.83	2.50
(N(1) + N(8))‐acetylspermidine	2.80	2.67
4‐Hydroxybenzyl alcohol	2.79	3.14
Uridine 5'‐monophosphate (UMP)	2.69	2.41
**Indolin‐2‐one**	**2.59**	**2.12**
(3′–5′)‐adenylylcytidine	2.45	1.72
Maleate	2.43	2.36
Alpha‐ketoglutarate	2.42	5.00
**Myo‐inositol**	**2.35**	**2.20**
2‐Hydroxyglutarate	2.34	2.23
**Indole‐3‐acetamide**	**2.33**	**2.45**
4‐Hydroxyphenylpyruvate	2.32	6.00
Arabitol/xylitol	2.31	1.99
5‐Hydroxymethyl‐2‐furoic acid	2.29	2.46
1‐Carboxyethyltyrosine	2.29	1.98
Homostachydrine	2.29	1.83
Guanine	2.29	0.31
1‐Carboxyethylvaline	2.27	1.85
Phosphothreonine	2.25	2.30
Mannose	2.25	2.38
3‐Methoxytyramine	2.24	2.15
Isocitric lactone	2.21	2.22
**Thioproline**	**2.20**	**1.71**
**1‐Kestose**	**2.18**	**1.00**
Glutarate (C5‐DC)	2.18	2.27
(3′–5′)‐uridylylguanosine	2.16	1.74
**Alpha‐hydroxyisovalerate**	**2.16**	**1.82**
*N*‐acetylhistamine	2.13	2.39
N‐acetylhistidine	2.08	1.71
(3′–5′)‐uridylyluridine	2.05	1.82
1‐Carboxyethylleucine	2.03	1.72
Allo‐threonine	2.01	0.96
Alpha‐ketoglutaramate	2.00	2.21
2′‐*O*‐methyluridine	1.97	1.70
Isocaproylglycine	1.96	1.33
1‐Linoleoylglycerol (18:2)	1.95	1.00
1‐Carboxyethylisoleucine	1.95	1.49
(3′–5′)‐cytidylylcytidine	1.93	1.20
Phosphoenolpyruvate (PEP)	1.91	1.00
*S*‐methylcysteine	1.90	2.04
3‐Hydroxy‐2‐methylpyridine	1.89	1.48
Creatinine	1.88	1.75
**Kynurenate**	**1.87**	**2.05**
3‐(4‐Hydroxyphenyl)propionate	1.82	1.84
7‐Methylguanine	1.82	1.70
*N*‐acetyltryptophan	1.80	1.86
Erythronate	1.80	1.79
**Choline phosphate**	**1.78**	**1.85**
Ribulonate/xylulonate/lyxonate	1.73	1.78
*N*‐acetylglutamine	1.73	1.46
*N*‐methylproline	1.72	0.92
*S*‐1‐pyrroline‐5‐carboxylate	1.71	2.02
**2,3‐Dihydroxyisovalerate**	**1.70**	**1.71**
Acetylagmatine	1.69	1.75
Methylsuccinate	1.67	1.60
Ribonate	1.66	1.67
Mannonate	1.65	1.68
Glycerophosphoinositol	1.63	1.57
Homocitrulline	1.61	1.83
*N*6‐acetyllysine	1.61	1.63
Glycerol 3‐phosphate	1.59	1.93
3‐Amino‐2‐piperidone	**1.57**	**1.57**
2‐Isopropylmalate	1.57	1.61
Threonate	1.54	1.67
1‐Methyl‐beta‐carboline‐3‐carboxylic acid	1.54	1.76
Cysteinylglycine	1.52	1.00
*N*‐acetylproline	1.52	1.35
3‐Methylglutaconate	1.52	1.40
Chiro‐inositol	1.51	1.30
Pentose acid	1.50	1.59
Imidazole lactate	1.50	1.39

a Some of the metabolites detected in the supernatant could be due to cell lysis.

b Fold difference in metabolites detected in culture supernatant compared to media control. Metabolites with potential health benefits are highlighted in bold.

**Table B5:** Top 50 *L. reuteri* 3632 proteins detected in the supernatant^a^

Identified proteins	Accession number	Molecular weight (kDa)	Spectra counts
**C protein alpha‐antigen precursor**	IVR12_00948	324	640
**Chromosome segregation protein**	IVR12_02229	115	419
Xylulose‐5‐phosphate phosphoketolase	IVR12_01032	91	375
6‐Phosphogluconate dehydrogenase, decarboxylating	IVR12_01054	53	254
**Chromosome partition protein Smc**	IVR12_02183	80	230
50S ribosomal protein L2	IVR12_00775	30	176
30S ribosomal protein S4	IVR12_01845	23	155
30S ribosomal protein S1	IVR12_00279	46	150
Enolase	IVR12_01715	48	142
**Hypothetical protein**	IVR12_01750	98	142
Fumarate hydratase class II	IVR12_00841	50	133
Elongation factor G	IVR12_00780	77	131
**d‐Gamma‐glutamyl‐meso‐diaminopimelic acid endopeptidase CwlS**	IVR12_02116	23	127
Glyceraldehyde‐3‐phosphate dehydrogenase	IVR12_01712	36	126
Citrate lyase alpha chain	IVR12_00832	55	119
Phosphoglycerate kinase	IVR12_01713	43	119
**Hypothetical protein**	IVR12_00140	31	118
Trigger factor	IVR12_00506	49	113
**Hypothetical protein**	IVR12_02192	169	112
60 kDa chaperonin	IVR12_01668	57	108
DNA‐directed RNA polymerase subunit beta	IVR12_00786	135	106
Pyruvate kinase	IVR12_00401	52	106
30S ribosomal protein S7	IVR12_00781	18	105
50S ribosomal protein L15	IVR12_00759	15	104
Isoleucine–tRNA ligase	IVR12_00558	109	102
50S ribosomal protein L6	IVR12_00763	20	102
Citrate lyase subunit beta	IVR12_00833	33	101
Alcohol dehydrogenase1	IVR12_00790	36	100
Elongation factor Ts	IVR12_00463	32	100
50S ribosomal protein L19	IVR12_02459	15	99
Valine–tRNA ligase	IVR12_01849	102	98
50S ribosomal protein L3	IVR12_00778	24	95
Putative amino‐acid ABC transporter‐binding protein precursor	IVR12_01610	29	86
Arabinogalactan endo‐1,4‐beta‐galactosidase precursor	IVR12_00642	166	85
Maltose phosphorylase	IVR12_01307	87	85
GTP‐binding protein TypA/BipA	IVR12_00521	69	85
Chaperone protein DnaK	IVR12_00443	67	84
50S ribosomal protein L13	IVR12_00746	16	84
Glucose‐6‐phosphate 1‐dehydrogenase	IVR12_01053	56	82
50S ribosomal protein L14	IVR12_00768	13	82
30S ribosomal protein S5	IVR12_00761	18	81
30S ribosomal protein S8	IVR12_00764	15	79
DNA‐directed RNA polymerase subunit beta'	LREU3632_00816	82	77
Alanine–tRNA ligase	IVR12_01878	99	77
30S ribosomal protein S9	IVR12_00745	14	74
50S ribosomal protein L11	IVR12_01623	15	72
30S ribosomal protein S19	IVR12_00774	10	71
50S ribosomal protein L5	IVR12_00766	20	70
Elongation factor P	IVR12_00896	21	70
30S ribosomal protein S3	IVR12_00772	25	69

^a^ Some of the proteins detected in the supernatant could be due to cell lysis.

**Table B6:** Top 50 proteins detected in *L. reuteri* 3632 cell pellet

Identified proteins	Accession number	Molecular weight (kDa)	Spectra counts
**Xylulose‐5‐phosphate phosphoketolase**	IVR12_01032	91	1422
**Elongation factor Tu**	IVR12_00507	43	1196
Arabinogalactan endo‐1,4‐beta‐galactosidase precursor	IVR12_00642	166	1059
Aldehyde‐alcohol dehydrogenase	IVR12_01636	97	869
DNA‐directed RNA polymerase subunit beta	IVR12_00786	135	612
6‐Phosphogluconate dehydrogenase, decarboxylating	IVR12_01054	53	609
50S ribosomal protein L2	IVR12_00775	30	565
**Glyceraldehyde‐3‐phosphate dehydrogenase**	IVR12_01712	36	557
Enolase	IVR12_01715	48	480
DNA‐directed RNA polymerase subunit beta'	LREU3632_00816	82	469
Alcohol dehydrogenase 1	IVR12_00790	36	429
30S ribosomal protein S3	IVR12_00772	25	413
3‐Oxoacyl‐[acyl‐carrier‐protein] synthase 2	IVR12_02345	77	392
**C protein alpha‐antigen precursor**	IVR12_00948	324	377
30S ribosomal protein S1	IVR12_00279	46	375
Ornithine carbamoyltransferase	IVR12_01773	38	372
Elongation factor G	IVR12_00780	77	360
30S ribosomal protein S2	IVR12_00464	30	360
**Pyruvate kinase**	IVR12_00401	52	347
Maltose phosphorylase	IVR12_01307	87	331
30S ribosomal protein S4	IVR12_01845	23	318
Asparagine synthetase [glutamine‐hydrolyzing] 1	IVR12_01236	75	315
Trigger factor	IVR12_00506	49	311
Citrate lyase alpha chain	IVR12_00832	55	307
GTP‐binding protein TypA/BipA	IVR12_00521	69	302
Fumarate hydratase class II	IVR12_00841	50	300
Nuclease SbcCD subunit C	IVR12_02566	119	300
60 kDa chaperonin	IVR12_01668	57	292
Glucose‐6‐phosphate 1‐dehydrogenase	IVR12_01053	56	283
Elongation factor Ts	IVR12_00463	32	278
50S ribosomal protein L3	IVR12_00778	24	271
Phosphoglycerate kinase	IVR12_01713	43	268
**l‐lactate dehydrogenase**	IVR12_00437	34	266
Translation initiation factor IF‐2	IVR12_00451	77	260
30S ribosomal protein S5	IVR12_00761	18	253
Putative amino‐acid ABC transporter‐binding protein	IVR12_01610	29	252
50S ribosomal protein L5	IVR12_00766	20	247
30S ribosomal protein S8	IVR12_00764	15	246
1,3‐propanediol dehydrogenase	IVR12_01279	42	244
Alanine–tRNA ligase	IVR12_01878	99	242
30S ribosomal protein S13	IVR12_00754	14	241
50S ribosomal protein L14	IVR12_00768	13	240
50S ribosomal protein L15	IVR12_00759	15	237
Putative ATP‐dependent Clp protease ATP‐binding subunit	IVR12_01298	82	236
50S ribosomal protein L18	IVR12_00762	13	235
Diacetyl reductase [(S)‐acetoin forming]	IVR12_00239	28	232
30S ribosomal protein S10	IVR12_00779	12	230
Recombinase A	IVR12_01868	39	230
30S ribosomal protein S19	IVR12_00774	10	224
Threonine–tRNA ligase	IVR12_01160	69	222

## APPENDIX C

Selection of final *L. reuteri* candidates delivering nanobodies against NetB and α toxin.

Colonization of engineered *L. reuteri* candidates.

### Materials and methods

Colonization of engineered *L. reuteri* candidates delivering different Nb clones against NetB and α toxin was determined as described in the materials and methods section.

### Results

Along with their respective parent strains, NE01 and NE06 were evaluated for colonization in chicken after in ovo administration. As shown in Figure C1, the mean CFUs/g of cecal contents ± SD for *L. reuteri* 3630 and 3632 were 8.36E + 06 (±4.14E + 06) and 1.07E + 07 (±4.49E+06), respectively. Similarly, the mean CFUs/g of cecal contents (±SD) for *L. reuteri* 3630 delivering EAT‐1G4 (NE01), *L. reuteri* 3630 delivering EAT‐1F2_R27H, *L. reuteri* 3630 delivering ENB‐1D11_R56H, *L. reuteri* 3630 delivering ENB‐1A4, and *L. reuteri* 3632 delivering ENB‐1D11_R56H (NE06) were 1.10E+07 (±1.42E + 06), 4.70E+06 (±3.87E+06), 1.11E+07 (±3.90E+06), 8.42E+06 (±6.06E+06), and 4.97E+ 06 (±1.39E+06) CFUs, respectively Figure C1. The mean CFUs of all the four strains were found to be not significantly different from each other (*p* > 0.05). These data suggest that all the tested engineered strains exhibit similar colonization levels to their respective parent strains (Figure C1).

Screening of engineered *L. reuteri* candidates for efficacy.

### Materials and methods

Efficacy study was conducted at Colorado Quality Research and the study was reviewed and approved by Elanco Animal Health Inc. Animal Care and Use Committee (IACUC# 1205). Two hundred and forty Cobb 500 commercial male broiler chicks were allocated to six groups with each containing 40 birds per group (10 birds per cage). Chicks from each replicate were housed in the same cage and replicates of the same group were housed on the same rack. The birds were housed and cared for according to the Guide for the Care and Use of Agricultural Animals in Research and Teaching. The birds were provided with ad libitum feed and drinking water. The feed ration included a commercial‐type broiler diet formulated to meet or exceed requirements stipulated by the National Research Council (Council, 1994).

The *L. reuteri* experimental products were prepared exactly as described in the “Materials and methods” section. *L. reuteri* candidates were administered in drinking water every day for 21 days at a dose of 1.0 × 10^8^ CFUs/bird (Table C1). Each day, vials containing the experimental product were resuspended with the appropriate volume of non‐chlorinated distilled water. Following resuspension, an aliquot was used for titration. Full‐day water requirement was divided into four equal parts. Each part was provided to one cage of the same treatment group. A similar procedure was applied to all the treatment groups from T2 to T6. Medicated water (containing *L. reuteri* experimental products) was provided to the respective treatment group birds for 24 h as ad libitum.

The NE model used in the present study includes oral administration of a mixture of *E. maxima* and *E. acervulina* followed by serial oral administration of *C. perfringens*. At Day 14 of age, the birds in Groups 2, 3, 4, 5, and 6 were challenged with 10,000 sporulated oocysts of *E. maxima–E. acervulina*/bird. The challenge inoculum was administered via oral gavage using a 10 ml syringe fitted with an 18‐gauge gavage needle.

Fresh *C. perfringens* (strain CL‐15, Type A, α and β2 toxins) challenge inoculum was prepared every day from a stock culture in fluid thioglycollate broth overnight at 35°C under anaerobic conditions by Microbial Research Inc. *C. perfringens* was administered by oral gavage at a target dose of approximately 1 × 10^8^ CFUs/ml (1 ml/chick) on 17, and 18 days of age.

Mortality and lesion scores were used as the primary criteria for efficacy evaluation. Chickens that died post‐challenge phase between 18 (after challenge with *C. perfringens*) and 22 days of age were necropsied, and the cause of death was listed as NE‐related or non‐NE‐related mortality. Lesion scores were performed on five birds per cage on Study Day 20. The birds were scored using the procedure described by Shojadoost et al. (2012).

### Results

As shown in Table C1, while Group 1 (no challenge control) had no NE‐associated mortalities, Group 2 (challenge control) showed 26.8% NE‐associated mortality and an NE lesion score of 1.8 (Table C1). Treatment with *L. reuteri* 3630 delivering EAT‐1G4 (NE01, Nb against α toxin) administered via drinking water at 1.0 × 10^8^ CFUs/bird/day (Group 3) significantly reduced NE‐associated mortality to 10.0% and NE lesion score to 0.50 (Table C1; *p* < 0.05 compared to Group 2). Treatment with *L. reuteri* 3630 delivering EAT‐1F2_R27H (Nb against α toxin) administered via drinking water at 1.0 × 10^8^ CFUs/bird/day (Group 4) numerically reduced NE‐associated mortality to 19.5% and NE lesion score to 0.95 (Table C1; *p* > 0.05). Similarly, treatment with *L. reuteri* 3630 delivering ENB‐1A4 (Nb against NetB) administered via drinking water at 1.0 × 10^8^ CFUs/bird/day (Group 5) numerically reduced NE‐associated mortality to 22.5% and NE lesion score to 1.45 (Table C1; *p* > .05). Treatment with *L. reuteri* 3630 and 3632 each delivering ENB‐1D11_R56H (NE06, Nb against NetB) administered via drinking water at 1.0 × 10^8^ CFUs/bird/day (Group 6) significantly reduced NE‐associated mortality to 15.0% and NE lesion score to 0.70 (Table C1; *p* < 0.05 compared to Group 2). These data suggest that the administration of NE01 and NE06 in drinking water daily for 21 days protects chickens from NE. Based on these findings, NE01 and NE06 were used for further efficacy studies (Table C1).

**Table C1:** Efficacy evaluation of *L. reuteri* candidates delivering nanobodies against NetB and α toxin

Group	Treatment group	Dose/bird	Route of administration	NE % mortality	NE lesion score	% reduction in NE mortality compared to challenge control	% reduction in NE lesion score compared to challenge control
1	No challenge control	N/A	N/A	0.0	0.00		
2	Challenge control	N/A	N/A	**26.8**	**1.80**		
3	*Limosilactobacillus reuteri* 3630 delivering EAT‐1G4 (NE01)	1 × 10^8^ CFU/bird/day	Drinking water, daily for 28 days	**10.0** *	**0.50** *	**62.6**	**72.2**
4	*L. reuteri* 3630 delivering EAT‐1F2_R27H	1 × 10^8^ CFU/bird/day	Drinking water, daily for 28 days	19.5	0.95	27.2	47.2
5	*L. reuteri* 3630 delivering ENB‐1A4	1 × 10^8^ CFU/bird/day	Drinking water, daily for 28 days	22.5	1.45	16.0	19.4
6	*L. reuteri* 3630 delivering ENB‐1D11_R56H & *L. reuteri* 3632 delivering ENB‐1D11_R56H (NE06) in 1:1 ratio	Combined dose, 1 × 10^8^ CFU/bird/day	Drinking water, daily for 28 days	**15.0** *	**0.70** *	**44.0**	**61.1**

*Note*: Highlighted in bold, **p* < 0.05 compared to Group 2.

Abbreviation: NE, necrotic enteritis.
